# Bioinformatic and computational biophysics tools for nanopore engineering: a review from standard approaches to machine learning advancements

**DOI:** 10.1186/s12951-026-04225-4

**Published:** 2026-03-05

**Authors:** Marco Reccia, Francesco Quilli, Kherim Willems, Blasco Morozzo della Rocca, Domenico Raimondo, Mauro Chinappi

**Affiliations:** 1 Department of Industrial Engineering, University of Rome Tor Vergata, Via del Politecnico 1, Rome, 00133 Italy; 2 Department of Molecular Medicine, Laboratory Affiliated to Istituto Pasteur-Fondazione Cenci Bolognetti, Sapienza University of Rome, Viale Regina Elena 291, Rome, 00133 Italy; 3 imec, Kapeldreef 75, Leuven, 3001 Belgium; 4 Department of Biology, University of Rome Tor Vergata, Via del Politecnico 1, Rome, 00133 Italy

**Keywords:** Nanopores, Mutations, Protonation state, APBS, Molecular dynamics, Protein structure prediction, De novo design

## Abstract

Nanopores are key components in several emerging technologies. Nanopore sensors allow the observation of single molecules from the signature they leave in the ionic current flowing through the pore, while nanoporous membranes provide a potentially customizable platform to achieve unprecedented ion selectivity performance. In parallel to the improvements in the fabrication techniques, computational methods have flourished in the last few years. However, despite these advancements, bioinformatic and computational biophysics tools are still not systematically employed in nanopore research when compared to other engineering fields that integrated computer-assisted design (CAD) in their development pipeline decades ago. This review aims to provide a wide-ranging overview of the main bioinformatic tools useful for the engineering of biological nanopores including: analysing the effect of mutations on pore properties, determining the protonation state at different pH and studying the electrostatic environment via adaptive Poisson-Boltzmann solver (APBS). A final section presents recent progress in *de novo* design using AI-based methods. To favour the widespread adoption of these approaches, the Supplementary Information contains some scripts and protocols that may aid the readers to integrate these tools in their design approaches.

**Graphical Abstract:**

**Figure:**
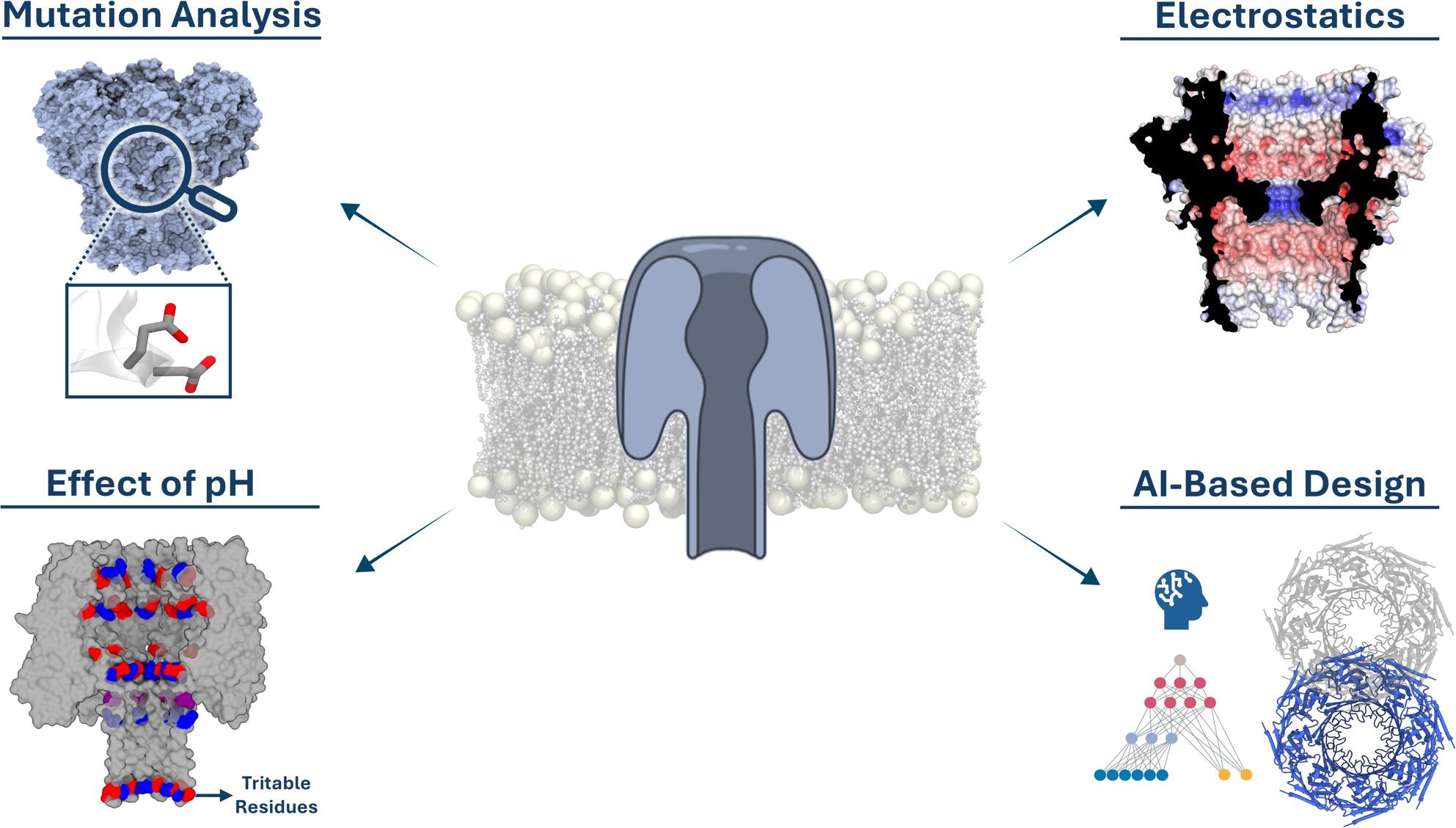
Schematic overview of nanopore features that can be investigated using bioinformatic tools.

**Supplementary Information:**

The online version contains supplementary material available at 10.1186/s12951-026-04225-4.

## Introduction

Nanopores are an established technology for single-molecule DNA sequencing, with the first commercial devices delivered a decade ago [1]. This success has led several research groups to explore the possibility of extending nanopore sensing to other classes of biologically relevant particles, including biomolecules (*e.g.*, proteins [2–8] and sugars [9, 10]), molecular aggregates [11–13] and entire viruses [14]. Nanopore sensing is not limited to the characterization of particles in a sample. Several biologically relevant questions have been investigated, such as the rate of enzymatic reactions [15] and the conformational stability of proteins [16]. Moreover, nanopore technology is a promising candidate for breakthroughs in other fields, such as ultrafiltration [17], desalination [18], energy harvesting [19, 20], detection of heavy metal ions [21, 22] and data storage [23, 24].

In a typical nanopore sensor, a single nanopore is embedded in a membrane, where it connects two reservoirs containing an electrolyte solution. A voltage applied between the reservoirs generates an electric current (open pore current) due to the movement of ions through the nanopore. When particles are introduced in one compartment, the interaction between dispersed particles and the nanopore leaves a signature in the current trace that can be used to infer nanoparticle properties [25–28]. Both the open pore current and the particle signature strongly depend on the nanopore structure, which must be designed to enhance the sensor’s ability to distinguish different molecules. Capturing the particles to be analyzed is also an engineering challenge. Particles are dispersed in one of the two reservoirs, whose size is often several orders of magnitude larger than the pore entrance. For example, in biological nanopores such as MspA [29] or CsgG [30, 31], the diameter of the pore entrance is a few nanometers, while the reservoir’s typical length scale is at least hundreds of micrometers. When molecules are highly charged, such as DNA or RNA, the electrophoretic force can easily funnel them to the nanopore. However, for weakly charged molecules, capture remains challenging [32]. Proteins provide a notable example, as they do not have a homogeneous charge distribution and are usually weakly charged compared to nucleic acids. Consequently, electrophoresis is not always a viable capture strategy. In this context, electroosmosis (*i.e.*, the flow of liquid induced by the applied voltage [33, 34]) is the most promising approach. However, electroosmosis strongly depends on fine details of the surface charge distribution inside the nanopore and near its openings [5, 35].

Even limiting our analysis to the above-mentioned simplified scenario, it is clear how nanopore design is challenging, as the pore needs to be optimized for two properties: the ability to distinguish different molecules and a high electroosmotic flow to capture them. These two properties generally cannot be optimized independently, as both are largely dominated by the narrowest region of the nanopore [25, 35]. A further obstacle in nanopore design is represented by the limitations of fabrication techniques. Even if we theoretically or computationally determine the optimal shape and surface charge of the pore in order to get distinguishable signals and high capture rates, it is often not possible to control nanopore structure at the sub-nanometer scale.

Presently, the most reliable strategy to fabricate nanopores with prescribed shape and surface charge is to use as scaffolds existing biological channels. Examples are $\alpha$-hemolysin ($\alpha$HL) [36], aerolysin [37], Mycobacterium smegmatis porin A (MspA) [29], and Curli-specific gene product G (CsgG) [30] that are trans-membrane proteins constituted by repetition of the same protein chain (homo-multimers or homomers in short) and for which reliable structures are publicly available in the Protein Data Bank (PDB) [38]. Directed mutagenesis of a few residues is widely employed to alter the pore lumen and for some pores, such as MspA or a erolysin, tens of mutants have been analyzed [35, 39]. More elaborate approaches involve incorporating other biomolecules such as nanobodies or aptamers at the extremities of flexible chains to selectively retain specific analytes [40–42]. Other strategies include stacking several pores to form complexes able to enzymatically unfold (or cut) the translocating polymer [43], chemically altering one chain to promote specific binding reactions [44], and *de novo* design of pores [45, 46].

With a few exceptions, the majority of breakthrough achievements of the last few years was obtained by leveraging the physico-chemical intuition of researchers that smartly modified existing nanopores [2, 5, 35, 43, 47, 48]. In some cases, the initial intuition was supported by preliminary computational analysis, such as the *in silico* mutation of specific residues, the calculation of electrostatic properties [5] and molecular dynamics (MD) simulations [35, 49–51]. However, using such preliminary computational analyses to assist pore design is not as widespread as in other fields of engineering and chemistry, where computer-assisted design (CAD) is a well-established practice. In our view, this is a drawback of the intrinsic multi-disciplinarity of the burgeoning nanopore community, whose members are not necessarily aware of the state of the art of bioinformatic, computational biology and computational nanofluidics tools potentially useful for nanopore engineering.

This review aims to contribute to filling this gap. First, we review standard approaches already used in some nanopore studies, such as *in silico* mutagenesis, electrostatic calculation and protein modelling. We will not limit ourselves to reviewing the most common tools, but we will also apply them to some specific cases showing how different software can provide slightly different results, and we propose strategies to reduce possible bias. Moreover, in the supplementary information, we report several scripts and protocols used and that may be useful for the readers who are non-experienced in bioinformatics. Second, we survey the state of the art of standard structural bioinformatic tools that, although widespread in other biochemical applications, are not commonly used in nanopore studies and that, in our view, can be beneficial to the field. Examples are methods for the prediction of solvent accessibility and pore flexibility. Third, we discuss MD simulations. Since this topic was already the subject of other reviews [34, 52] and several tutorials are available [53, 54], we will limit our contribution to the discussion of some open challenges. Finally, we comment on recent works on *de novo* designed pores and on the role of artificial intelligence in nanopore design.

## Modifying known nanopore 3D structures

A quite common situation in nanopore studies is that the structure of a nanopore has been experimentally solved via X-ray or cryo-EM and made available to the community in the PDB [38]. In some cases, the structure is already complete and researchers only want to modify part of it to control, for example, the size of the constriction, the surface charge, or the hydrophobicity of the lumen. In other cases, the structure is incomplete and some missing fragments need to be rebuilt. In the following, we will review the main tools used to computationally support the design of modified nanopores starting from solved structures.

### Point mutation modelling

**Fig. 1:**
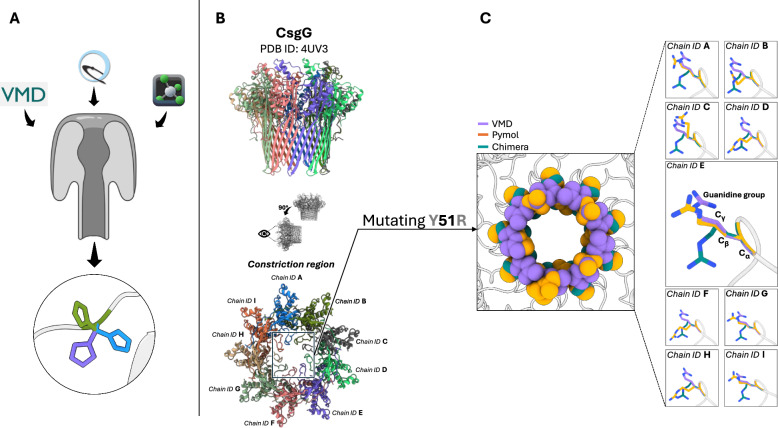
Differences in modeled side chains for point mutations. **(A)** Schematic representation of the process of *in silico* mutagenesis performed on a nanopore scaffold using three commonly employed molecular modeling tools, VMD, UCSF ChimeraX, and PyMOL. Following different approaches to side chain replacement, the internal algorithms generate distinct conformations for the same target mutation. **(B)** Structural context of the mutation site within the *Escherichia coli* curli secretion channel, CsgG (PDB ID: 4UV3 [30]). Top and side views of the nonameric pore highlight the $\beta$-barrel region encompassing the central constriction zone, where the Y51R substitution was modeled. Each chain is shown in a different color, and chain identifiers are labeled. The boxed region marks the constriction region, relevant for molecular sensing applications [55], where the mutation was introduced. **(C)** Comparison of the resulting arginine side chains modeled by VMD (purple), ChimeraX (teal), and PyMOL (orange). While the $\text {C}_{\alpha }$ atoms remain relatively aligned, differences in arginine side chain orientation begin at the $\text {C}_{\beta }$ atoms and extend through the $\text {C}_{\gamma }$ atoms and guanidinium group. These discrepancies were observed in all nine chains of the complex. Details on the protocols used to mutate the nanopores are reported in Supplementary Note S1

In the context of biological nanopore engineering, the substitution of single amino acids represents a key strategy for modifying the structural and functional properties of nanopores. Since the early days of nanopore sensing research, point mutations have been used to alter the pore properties. For instance, mutations in the $\alpha$HL constriction allows modulating the pore’s affinity for cyclodextrin [56] while negative residues in MspA have been mutated to allow the translocation of DNA [39]. A common route to achieve an electroosmotic flow strong enough to capture an analyte molecule, is the counterion accumulation in the pore lumen due to fixed surface charge [34]. Thus, several studies explored mutants carrying different side chain charge distributions in the pore lumen to control strength and direction of electroosmosis [8, 10, 35, 48, 57]. The alteration of the pore charge distribution also affects the electrostatic contribution to the free-energy barrier associated with the transport of charged and polar molecules as shown, for instance, for DNA translocation in MspA [39], for dsDNA translocation in Cytolysin A (ClyA) [58], for peptide capture in Fragaceatoxin C (FraC) [5], for protein capture by Pleurotolysin A (PlyAB) [57], for DNA and peptide sensing in [59] and for norfloxacin (a small polar molecule) in [60].

Accurate *in silico* modeling of protein mutations requires precise prediction of amino acid side-chain conformations, as they are intrinsically linked to protein stability, specificity, and function. In recent years, many successful software implementations have been proposed to address the side-chain modeling problem [61–71]. Conventional side-chain modeling methods are typically composed of three elements: i) a rotamer library that provides discrete conformational states, ii) a scoring function that evaluates conformational plausibility, and iii) a search algorithm that optimizes side-chain placement within the protein environment. These approaches offer high computational efficiency, often reconstructing full side-chain configurations within seconds. However, their reliance on discrete rotamer sampling constrains their predictive accuracy, as the conformational space is limited by the resolution and diversity of the rotamer library. Recent advances in deep learning have prompted the development of data-driven approaches that aim to transcend these limitations by learning continuous, context-aware side-chain representations directly from structural data, thereby improving accuracy and generalizability in side-chain modeling tasks [70, 71].

Here, with the purpose to show how standard tools may provide different predictions for side chain conformation, we compared three tools frequently employed to design mutants in the nanopore field: VMD [72], PyMOL [73], and UCSF ChimeraX [74, 75]. A key aspect that has emerged from the comparative study of these tools is the variability in side chain conformations generated for the same mutation. For instance, in the case of the Y51R, which we introduced in the nanopore CsgG (PDB ID: 4UV3 [30]), each tool generated a different conformation for the arginine residue, (Fig. 1).

This behavior is not specific of the CsgG nanopore: similar inconsistencies were observed for other mutations introduced at different positions within the nanopore or in other proteins analyzed (Fig. S1). These differences are presumably due to the distinct rotamer selection engines, which rely on varying strategies for conformational prediction. A concise comparative overview of rotamer selection strategies implemented in the different software packages is provided in Supplementary Table S1, which helps contextualize these divergences and serves as a guide to the methodological differences discussed below. In PyMOL, mutagenesis is handled through the “Mutagenesis Wizard”, which provides a list of rotamers derived from the Dunbrack Backbone-Dependent and Backbone-Independent libraries [76]. In this case, the graphical interface allows the user to directly visualize steric clashes, leading to a qualitative user-driven selection. In the case of automated use via basic commands from the Mutagenesis Wizard, the program automatically selects the rotamer with the highest probability according to the libraries, without additional structural evaluations. ChimeraX allows two operational modes: manual and automatic. In manual mode, the user can graphically view all available side chain rotamers and interactively select the desired one. Alternatively, the automatic mode selects a single rotamer based on a combination of criteria: best fit to the electron density map (if available), lowest steric clash score, highest number of hydrogen bonds formed, and highest probability according to the rotamer library (*e.g.*, Dunbrack), independently of the local environment but considering the dihedral angles of the protein backbone, $\phi$ and $\psi$. ChimeraX applies these criteria in a hierarchical order, using the next only in case of a tie according to the previous one. For example, if no electron density map is available (as always when using the tool to engineer a mutant from a wild type solved 3D structure), the program will evaluate steric clashes. If multiple rotamers show the same clash score, it proceeds to count hydrogen bonds, and finally to rotamer probability^1^. VMD, instead, does not natively include an interactive rotamer selection system like PyMOL or ChimeraX. Mutations can be introduced through the graphical interface (Mutator Plugin) or via Tcl scripting. VMD Mutator internally relies on psfgen (a VMD plugin for building molecular structures), which generates the new side chain using the spatial information specified in the topology file. There is no dynamic selection, energy scoring, or clash evaluation: the inserted rotamer is simply the one encoded in the topology. Details on the protocols we used to mutate the nanopores are reported in Supplementary Note S1.

The possible discrepancy in the mutant’s structures predicted using different tools, see Fig. 1C, calls for caution in their usage for nanopore design. First, we suggest to always try different tools and to verify if the predicted conformations are similar. Indeed, even if it is not possible to *a priori* exclude that some tools provided a wrong prediction of the configuration of the side chain, a consensus among them may be considered a robust indication on the reliability of the structures. Instead, if large differences are observed, we suggest to run an MD equilibration starting from all the structures and to check if after equilibration the differences persist. When molecular dynamics (MD) simulations are not feasible, mutant protein structures can be generated using comparative modeling tools such as MODELLER [77] or SWISS-MODEL [78]. These approaches allow the introduction of point mutations and, when required, the prediction of the entire mutant structure through homology modeling, often accompanied by energy minimization [5, 7, 79]. Alternatively, AI-based structure prediction methods may be employed to model mutant structures, as described in Sect. "De novo design of nanopores". To ensure the reliability of the generated models prior to MD simulations, a filtering strategy can be applied based on structural quality and stability criteria. This includes evaluation of global and local model quality metrics, such as DOPE (Discrete Optimized Protein Energy) scores from MODELLER [80] and QMEAN (Qualitative Model Energy ANalysis) [81] from SWISS-MODEL. In addition, careful inspection of the structural environment surrounding the mutation site, particularly the preservation of local secondary structure, can further inform model selection. Mutant models exhibiting unfavorable energy scores, severe steric clashes, or unrealistic backbone distortions are excluded, thereby reducing computational cost and focusing subsequent MD simulations on the most physically plausible structures.

### Effect of pH on surface charges

**Fig. 2:**
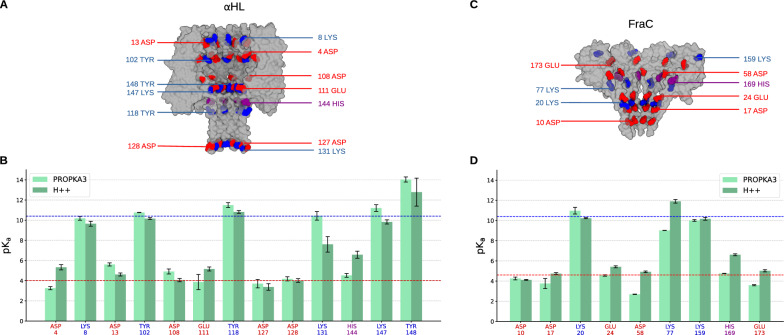
Effect of pH. **(A)** Surface representation of $$\alpha$$-Hemolysin (PDB ID: 7AHL [36]) cut along the axis. Titratable residues exposed toward the lumen are in blue (lysines and tyrosines), red (aspartates and glutamates) and purple (histidines). The comparison of predicted pK$$_a$$ values for these residues from PROPKA3 [82] and H++ [83] is in panel **(B)**. Each bar represents the pK$$_a$$ averaged over the monomers, with error bars indicating the standard deviation. The red and blue horizontal lines indicate examples of relevant acidic and basic pH for which a certain prediction of the protonation state is not possible (p$$K_a$$$$\simeq$$ pH, hence *d* from (3) is not close to 0, fully protonated, or 1, fully deprotonated). Panels **(C)** and **(D)** refer to FraC, PDB ID: 4TSY [84]. The complete lists of PROPKA and H++ predictions are in Supplementary table S1-S6

The pH is a central parameter in the study of biological nanopores, as it directly affects the protonation state of titratable amino acid residues in the protein structure [85–88]. Early studies on $\alpha$HL showed that the capture rate of neutral molecules was affected by the pH [86]. Indeed, the pH affects the protonation state of residues exposed towards the pore lumen. This alters the pore selectivity for cations (or anions) and, more in general, the net charge of the solution in the pore. The external electric field resulting from the application of a voltage between the two reservoirs acts on the mobile charges in the pore resulting in a force that drags the fluid, resulting in an electroosmotic flow (EOF) [34, 56, 89]. These early findings were then exploited by several authors [5, 87, 90–93]. For instance, Asandei et al.[93] showed that at pH=2.8, $\alpha$HL is able to capture short charged peptides even against the electrophoretic force. Similarly, a combination of pore mutation and pH control was used by Huang et al. [5] to capture peptides in a mutant of FraC independently of their charge, and to distinguish between hemoglobin mutants differing by only a single residue with a PlyAB mutant [94].

To model these effects at the atomic level (for instance, using MD) or to attempt a qualitative interpretation it is essential to assign the proper protonation states to the residues. The protonation state of a titratable residue is determined by the pH of the environment and the residue’s $pK_a$. Specifically, the $pK_a$ is related to the dissociation constant $K_a$ by

1 $$\begin{aligned} \textrm{p}K_a = -\log _{10}(K_a) \, , \end{aligned}$$

with

2 $$\begin{aligned} K_a = \frac{[\textrm{R}][\textrm{H}^+]}{[\textrm{HR}]} \, . \end{aligned}$$

where $\textrm{HR}$ and $\textrm{R}$ are the concentrations of the protonated and deprotonated forms of the residue, respectively. At a given pH, the fraction of deprotonation *d*, *i.e.*, the probability that a side chain is in its deprotonated form, can be calculated as

3 $$\begin{aligned} d = \frac{[\textrm{R}]}{[\textrm{HR}] + [\textrm{R}]} = \frac{K_a}{K_a + [\textrm{H}^+]} = \frac{1}{1 + 10^{pK_a - pH}} \, . \end{aligned}$$

This expression enables the estimation of the protonation state of each titratable residue. For example, for $pK_a = pH$ we have $d = 0.5$, meaning that, given an ensemble of titratable groups, half of them are protonated and half are deprotonated.

Several tools have been developed to predict the $pK_a$ given the protein structure [95–97]. Here, we compared the prediction of two commonly used ones, PROPKA3 [82] and H++ [83]. To test for possible discrepancies between the two predictive tools, we selected the residues exposed towards the pore lumen for $\alpha$HL (PDB ID: 7AHL [36]) and FraC (PDB ID: 4TSY [84]). Indeed, alteration of the charge of these residues is expected to affect the electrostatics in the pores with consequences on cation/anion selectivity and EOF. For PROPKA3 we used a locally installed version while for H++ the web server. In both cases, defaults parameters were used, see Supplementary Note S2 for details. Both tools provide a $pK_a$ for each titratable residue, so, in principle, even being homomers, the dissociation constants *d* from (3) for a specific residue may differ for each copy of a specific residue, presumably reflecting a not perfect symmetry of the original crystal structure. Fig. 2 reports the $pK_a$ averaged on the different chains for both pores.

In $\alpha$HL, we observed several discrepancies between PROPKA3 and H++, see Fig. 2B. For instance, for Glu111, PROPKA3 predicts $pK_a = 3.87$ while H++ gives $pK_a = 5.16$. Similar, or even larger difference is observed for, Asp13 (PROPKA3 5.6, H++ 4.6), His144 (PROPKA3 4.7, H++ 6.1) Asp4 (PROPKA3 3.2, H++ 5.3). Such differences may be relevant since they fall within a range of pH often used for $\alpha$HL [56, 91, 98–100]. Similar consideration holds for alkaline conditions. For instance, at pH=10.4 (Fig. 2B dashed blue line), several residues fall near or across their predicted deprotonation thresholds. For some of them, (Lys8, Tyr102), the predictions of both tools are similar, while for others (Lys131, Lys147) substantial differences are observed. A similar scenario occurs for FraC, Fig. 2C. For mildly acidic conditions around pH 4.5 Asp58 exhibits the largest discrepancy (PROPKA3: 2.7; H++: 4.9) followed by His169, Glu173, Asp17 and Glu24. For alkaline conditions, instead, a large difference in $pK_a$ prediction is observed for Lys77 (PROPKA3: 9.0; H++: 11.9). Remarkably, for some residues such as Asp10 and Lys159, both known for their relevance for EOF [5] and hydrophobic gating [50], the p$K_\textrm{a}$ predictions are in strong agreement between the two tools. For completeness, Supplementary Tables S2-S7 reports the output for both pores and both tools. In Supplementary Figure S2, we also report the dependence of the $pK_a$ predicted by H++ on the ionic concentration in the typical range used in nanopore experiments (0.15–4 M). The prediction are only slighly affected by the ionic concentration.

In addition to the discrepancies among different pK$_a$ prediction tools, there are two other, often underestimated, issues related to pH. The first is that, in the preliminary design of nanopores, it is useful to have a single prediction for the protonation state for a residue in the chain and not N different values, with N the number of monomers in the pore. As evident from Fig. 2, for some residues there is a large variability among the chains, see, *e.g.* PROPKA3 prediction for Glu111 for $\alpha$HL and for Asp17 for FraC, making the determination of the protonation state ambiguous. The rigorous approach to this issue is to average the dissociation constants *d* for each chain obtaining an mean value $\langle d \rangle$. This value can be directly used to define the protonation probability. A less rigorous approach, but often more direct, would be to calculate the average $\langle pK_a \rangle$ and then calculating *d* using Eq. (3). Usually, the two approaches provide very similar results if the variability of $pK_a$ for the same residue of different chain is small. The second issue is that, as already mentioned, it is possible that the pH selected for the experiments is close to pK$_a$ for some residues, implying that the probability of deprotonation is not close to 0 (fully protonated) or 1 (fully deprotonated). This may have large implication if electrostatic calculation like APBS or MD simulation will be performed after the determination of the protonation state. In APBS, a possible solution is to assign a fractional charge to residues [5]. In MD, one possibility is to protonate only residues of some of the chains and then check the robustness of the results to slight changes in the number of protonated residues as in [91]. Furthermore, when available, high-resolution structural data, such as cryo-EM [101] or NMR [102], can provide ground-truth evidence of protonation states by revealing specific hydrogen bond networks not accessible to lower resolution structures.

As a final comment on protonation states, it is possible, (although not frequent) that titratable residues are exposed toward the membrane and not toward the pore lumen. These occurrences are to be treated with caution, as there are indications [103] that membrane insertion (a process well beyond the scope of the present review) can contribute to stabilize the neutral forms. There is a complex interplay between a number of factors (*i.e.,* membrane composition and features, depth of insertion,structure of the peptide fragment) but simulations showed drastic local $pK_a$ changes (up to $3 \Delta pK_a$) with respect to the fully solvated case [104]. An example relevant in nanopores is the residue E237 in Aerolysin, a titratable residue present in the pore barrel that faces the membrane lipids. In MD simulations [105, 106], this residue was patched as protonated. The rationale of this choice was recently supported by the high-resolution cryo-EM structure of Aerolysin in lipid environment, suggesting an interaction of protonated E237 with Q263, (see [107], Fig. 1f and Fig. S9). Since incorrect titration states can have both local and long-range effects and can impact the stability of the MD simulation systems, when cryo-EM structures are available they can be conveniently used to deduce the correct charge assignments, thanks to the different scattering amplitudes of neutral and charged atoms [108].

## Predicting pore properties

When the structure of a nanopore is available, computational tools can be exploited to extract physicochemical information. Such analyses provide insights into properties that are crucial for sensing performance and for guiding experimental design. In this section, we present a set of computational approaches aimed at predicting key physicochemical properties of nanopores, starting from their 3D-structure. These properties include the electrostatic environment, which is essential to formulate hypotheses on ion selectivity and electroosmotic flow, the geometry of the lumen, and the conformational flexibility of the pore. We also discuss the role of molecular dynamics simulations, but, rather than providing a full review, we highlight two specific open issues, role of force field and connection with experiments. To the best of our knowledge, these aspects have not been adequately discussed in previous dedicated reviews [34, 52] and/or tutorials [53, 54].

**Fig. 3:**
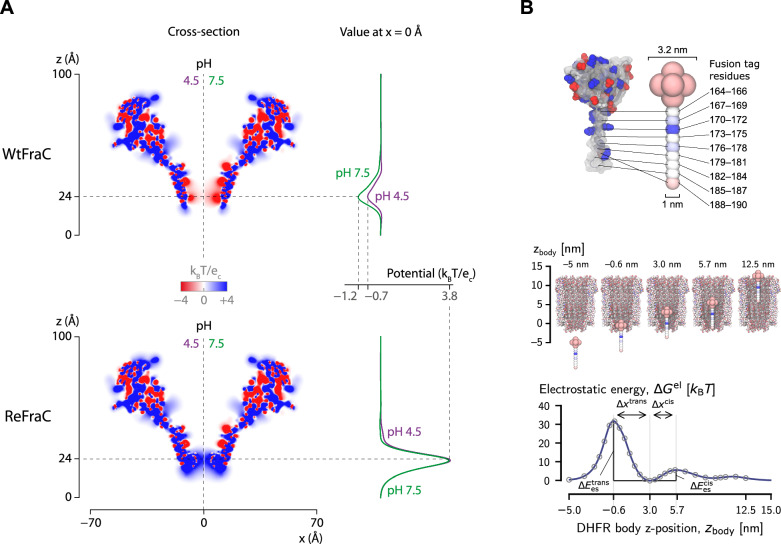
APBS for nanopores. **A** Impact of mutations and pH on the electrostatic fields in potential sliced through wild-type FraC (WtFraC, top) and the D10R-K159E-FraC (ReFraC, bottom) at pH 4.5 and 7.5. The Left-side panels report a slice-through heatmap of the electrostatic potential, and right-side panel represents its value along the central pore axis. Adapted with permission from [5]. **B** Example of how APBS can be used to calculate the electrostatic energy landscape of a translocating analyte. Panels show the coarse-grained model of tagged DHFR (top) as it is moved gradually through the nanopore ClyA, calculating at each step the electrostatic binding energy (bottom). Adapted with permission from [109]

### Surface charge, surface potential and selectivity

**Fig. 4:**
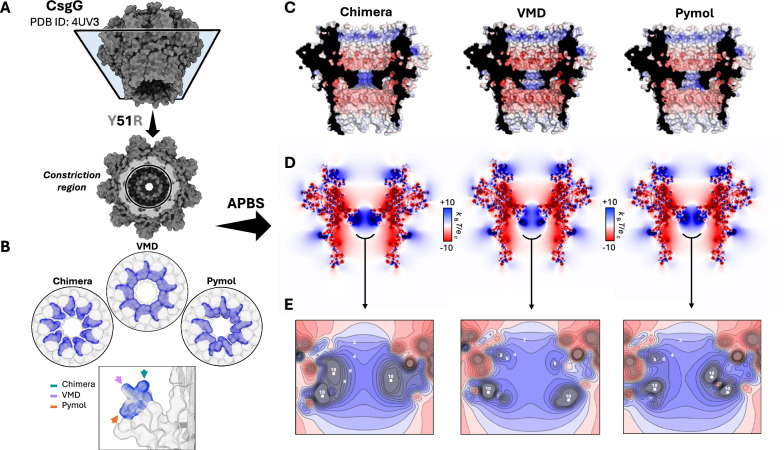
Impact of Y51R modeling approaches on the electrostatic potential of CsgG. **(A)** Surface representation of the CsgG nanopore (PDB ID: 4UV3) with a horizontal cross-sectional cut (highlighted in light blue) revealing the central constriction region. **(B)** Top-down view of the constriction, indicating the position of the Y51R mutation. The three panels compare how the introduced arginine residues (blue) are modeled in Chimera, VMD, and PyMOL, underscoring tool-specific differences in side chain orientation and surface rendering. A close-up of the mutation in one of the nine CsgG chains further illustrates variations in side chain conformation and cavity occupancy. **(C)** Electrostatic surface potential maps of the Y51R models, computed with APBS and projected onto the structures mutated by each modeling tool. Blue and red denote regions of positive and negative potential, respectively. **(D)** Corresponding 2D cross-sectional potential maps derived from the models in panel C. These maps emphasize how previously discussed variations lead to detectable changes in the local electrostatic profile across the pore constriction. **(E)** Kernel density estimate (KDE) plots of the constriction region, displayed with isolines spaced at 1 k$_B$T/e. This finer-grained representation quantitatively resolves subtle charge distribution differences introduced by the modeling tools, capturing distinctions not readily apparent in the broader 2D maps.

Electrostatics is a central aspect of nanopore function, as it controls ion selectivity, molecular capture, and electroosmotic flow. To get a preliminary description of electrostatics, the Poisson-Boltzmann (PB) equation is often employed. PB is a continuum model that corresponds to the equilibrium solution of the more general Poisson-Nernst-Planck or Poisson-Nernst-Planck-Stokes [34, 110] and, hence, it relies on several simplifying assumptions. Specifically, ionic concentrations are described as continuous charge densities following a Boltzmann distribution, the solvent is modeled as a uniform dielectric, and interactions are limited to mean-field electrostatics. While these assumptions are critical at the nanometer scale, PB calculations are extremely fast compared to atomistic simulations and thus provide an efficient first tool to probe the electrostatic environment of nanopores. PB is commonly used to model the electrostatic interactions in biomolecular systems, and it is typically solved using numerical approaches, such as finite difference or finite element methods. The adaptive Poisson-Boltzmann solver (APBS) [111, 112] is a widely used tool for solving the PBE in 3D biomolecular systems. APBS calculates the distribution of the electrostatic potential ($\phi$) and the corresponding local ion concentrations ($c_i$) in the electrolyte. These quantities can then be used to qualitatively assess the impact of mutations on the electrostatic field in the pore lumen and, consequently, to formulate hypotheses on ion selectivity and the electro-osmotic flow [5, 58]. Furthermore, APBS can also calculate the (a)polar solvation ($\Delta G$) and binding ($\Delta \Delta G$) energies, which allow to quantitatively estimate the Coulombic energy landscape of translocating analytes such as proteins [109] or DNA [113].

In practical terms, APBS requires two inputs: (1) atomic coordinates, radii, and partial charges of the biomolecular system (the “PQR” file), and (2) a set of parameters defining the PBE calculation (the “input” file). The PQR file can be generated directly with the PDB2PQR tool from any PDB file [114]. It assigns partial charges and radii based on the parameters of user-defined MD forcefields (*e.g.*, Amber, CHARMM, etc.), and assigns protonation states for all titratable residues at any given pH using their estimated pK$_a$ values as calculated by PROPKA [82]. When desired, PDB2PQR can also manipulate the placement of hydrogen atoms and the rotation of sidechains as to (naively) remove steric clashes and to optimize the hydrogen bonding network. Details for these procedures can be found in the PDB2PQR documentation [115]. The APBS input file (see Supplementary Note S3) is a simple text file that contains all the parameters required to set up and run the PBE calculation. These include, among others, the relative permittivities of the solvent ($\epsilon _r^s = 80$ for a water solution) and the protein ($\epsilon _r^p = 10$), the size, charge number and concentration for each ionic species, and the boundary conditions and grid parameters. A detailed description of all the settings and parameters can be found in the APBS documentation [116]. To assess the robustness of the calculated electrostatic potentials with respect to some of the key APBS parameters, we performed a sensitivity analysis to provide an overview of their impact. Full details are provided in the Supplementary Materials (Supplementary Figure S4). In brief, we suggest to use a grid space of 1Å. Moreover, the membrane thickness and the membrane dielectric constant do not significantly affect the electrostatic potential along the pore axis.

Finally, in an experimental setup, the biological nanopores are embedded within a thin lipid bilayer with a hydrophobic core that is both inaccessible to ions and has a low polarizability; characteristics that contrast strongly with those of the solvent. In ion channels, the membrane has a profound impact on the energetics of ion translocation [117]. Instead, for typical biological nanopores, with their larger water-filled channels and thicker sidewalls, ion desolvation effects are expected to play a much smaller role. Nevertheless, the explicit inclusion of the lipid bilayer provides at the very least a more realistic environment for calculating the electrostatic potential and energies. With APBS, the membrane can be explicitly taken into account in the calculation by modifying the dielectric and ion accessibility grids with the draw_membrane2 utility provided with APBS [118] to include a thin ($\approx 3$ nm [119] ion-inaccessible slab with a low permittivity ($\epsilon _r^m = 2$ [120]). More details can be found in the Supplementary Note S3. Note that while APBS treats protein permittivity as a constant value, typically in the range of 2–20, at atomic scale it is a spatially varying property that depends on the local protein and solvent density, polarizability of the side chains, and ionic strength [121].

APBS was been used to estimate the effect of pore mutations and buffer conditions (*i.e.*, pH and ion strength) on nanopore ion selectivity [5, 58] and energetics of analyte translocation [109, 113, 122, 123]. As an example, Huang et al. [5] used APBS to support the design of a specific mutant of the FraC nanopore (named ReFraC) and to suggest that working at pH 4.5 would have a major impact on nanopore anion selectivity and electroosmotic flow, (Fig. 3). APBS was also used to map the electrostatic energy landscape of a coarse-grained version of a tagged dihydrofolate (DHFR) enzyme translocating through ClyA. This provided both mechanistic insights into the trapping mechanisms (*i.e.*, the presence of a double energy well) and quantitative values of the energy barriers to aid in the fitting of an analytical model to the experimental data [109].

As a further example of the application of APBS to nanopores, we evaluated whether the mutations we previously introduced in CsgG and MspA (Sect. "Point mutation modelling") could alter the electrostatic properties of these pores. Given the heterogeneous positioning of the modeled side chains, particular attention was placed on the constriction region, where even subtle perturbations may affect pore function. To this end, we used a three-stage bash workflow: first, generating the PQR file, and then performing a preliminary APBS dummy run followed by a full run to compute the electrostatic potential. The split procedure is detailed in Supplementary Note S3, where both the complete workflow and simplified one-line commands are provided for reproducibility. The resulting calculations showed that the global electrostatic landscape of both pores remained largely unchanged (Fig. 4, Fig. S3), whether visualized in 3D by mapping the potential onto the pore surface or in 2D using z-slice representations. However, when focusing on the mutated region, corresponding to or in close proximity to the constriction site, subtle but consistent variations emerged. The alternative orientations and side-chain placements introduced by mutagenesis produced localized modifications in the electrostatic potential profile. These alterations are already discernible in the 2D z-slice plots, but are more clearly resolved in the KDE representation with isolines spaced at 1 k$_B$T/e (Fig. 4E, S3E). Although modest relative to the overall pore electrostatics, these shifts nonetheless reveal localized perturbations in the charge environment that may contribute to shaping pore functionality.

The PBE describes equilibrium electrostatics but does not provide non-equilibrium properties like ionic conductance or electro-osmotic flow. To model these, time-dependent methods are necessary, such as MD (see Sect. "Molecular dynamics") or continuum models like the coupled PNP-S equations [33, 34, 124]. While computationally cheaper than MD, PNP-S models require a detailed nanopore geometry and charge distribution. Their validity at the nanoscale also depends on non-trivial corrections for effects like finite ion size, nonlocal electrostatics, and local variations in ion diffusivity and conductivity. These models have been successfully used to predict ion and water transport for various biological nanopores, including ClyA [110, 125], PlyAB [57, 94], $\alpha$HL [126, 127], MspA [127], and CsgG [127].

### The size of the pore

The size of the pore lumen, and, in particular the presence of constrictions, is a relevant issue in the design of nanopore sensors, as constrictions are the regions that provide the largest contribution to the pore electric resistance. Simple continuum quasi-1D models, frequently complemented by models for the access resistance, are often used to get an idea of nanopore electric resistance and of the intensity of the external electric field due to the applied voltage [2, 128, 129]. Usually, these continuum models take as input the shape of the pore described in terms of the section *A* (or, more often the effective radius $r=(A/\pi )^{1/2}$) available to the transport of the electrolyte solution as a function of the axial coordinate *z*. Moreover, despite continuum quasi-1D models are not expected to provide quantitatively accurate results for narrow biological nanopores, quantifying the internal volume of nanopores is often employed to qualitatively interpret ionic current blockages [130, 131]. The determination of the section of the pore is conceptually and technically related to studies of protein cavities and tunnels, for which a variety of computational tools have been developed. Among the most widely used are CAVER [132], HOLE [133], and POVME [134]. These programs differ in their underlying algorithms, targeted applications, and the nature of the structural insights they provide. Some were developed for small cavities or for ionic channels, and they are not simply adapted to large nanopores. A quite flexible tool is POVME, that allows the definition of custom three-dimensional inclusion regions (such as cylinders, boxes, or spheres) and uses a grid-based method to calculate the volume accessible to a small probe within that region. Although originally developed to monitor dynamic pocket volumes in drug discovery, POVME 3.0 proved ideal for our single-frame analysis, as it enabled precise definition of cylindrical regions encompassing the full pore lumen from the extracellular to the intracellular side. As an example, we report in Supplementary Fig. S5 the application of POVME to CsgG and MspA. In both cases, the software efficiently captured the internal geometry of the nanopores, allowing us to quantify how subtle structural rearrangements influenced the lumen profile. The resulting equivalent-radius representations provided a clear means to discern even minimal variations in the pore’s internal space.

### Predicting pore flexibility

To date, to the best of our knowledge, no studies have been specifically dedicated to analyzing or predicting the flexibility of biological nanopore protein structures. Yet, nanopore flexibility plays a crucial role, as fluctuations in the 3D structure can influence ionic current noise and gating behavior [135–138]. Experimental determination of structural rigidity can be inferred from indicators such as B-factors in X-ray crystallography, local resolution in cryo-EM maps, or conformational ensembles in NMR data. However, for *in silico* designed novel nanopores or mutant variants, such data are unavailable, making predictive approaches indispensable. Experimental methods, including X-ray crystallography, NMR, and hydrogen–deuterium exchange coupled to mass spectrometry, remain resource-intensive and low-throughput [139], underscoring the need for scalable computational solutions. Importantly, local flexibility may also complicate structural prediction, since conformations considered “incorrect” could represent alternative, biologically relevant states. Computational methods thus offer valuable tools to assess flexibility, distinguishing between local residue-level motions and larger-scale rearrangements such as loop or domain movements. Molecular dynamics (MD) simulations, for example, allow quantification of regional flexibility via root-mean-square fluctuations (RMSF), as shown for aerolysin [122]. Nevertheless, MD is computationally demanding and difficult to standardize across studies, limiting its scalability as the number of nanopore structures grows with advances in protein prediction and *de novo* design, see Sect. "De novo design of nanopores". Alternative bioinformatic approaches can provide more rapid, if lower-resolution, insights: elastic network models and normal mode analysis efficiently capture collective motions but miss fine local loop dynamics [140, 141], while disorder predictors such as IUPred2A [142, 143] identify unstable regions without distinguishing flexible from rigid coils. Recently, AI-driven generative approaches have emerged as promising tools to explore ensembles of nanopore conformations [144]. In summary, although no standardized framework exists for nanopore flexibility prediction, established computational methods from structural bioinformatics, applied judiciously, can provide valuable preliminary insights and may guide future high-throughput analyses [145].

### Accessibility to the solvent

Solvent surface accessibility [146] is a basic property that indicates which residues are exposed to the lumen and can interact with analytes or undergo post-translational chemical modification. To the best of our knowledge, no systematic studies have applied solvent accessible surface area (SASA) analysis to nanopores. However, this quantity can be directly relevant for guiding mutagenesis and post-translational functionalization to add specific features to nanopores. Several examples illustrate this point. For instance, Boersma and Bayley introduced a cysteine at position 117 of $\alpha$-hemolysin and covalently attached a phenanthroline group that coordinates Cu(II), thereby creating a chemically reactive site within the pore lumen [147] and successively implemented a series of sequential modifications along the pore to realize a “hopping track” to move molecules in steps [148]. A number of examples of mutations of pore lining residues to exploit their differential interaction with substrates or ions involve cysteine, histidine or charged residues [149–151], or hydrophobic ones [47, 152]. More complex chemistry can be built upon initial mutations, provided that the ”handle" is accessible to the solvent, as in [153–155]. Concerning the available tools, solvent accessible surface area can be readily calculated from a PDB structure using widely adopted programs such as DSSP [156], NACCESS [157], FreeSASA [158] or VMD [72]. These methods only require the three-dimensional coordinates of the protein (e.g., a PDB file) and do not need additional input, making them fast and accessible for routine analysis.

### Molecular dynamics

**Fig. 5:**
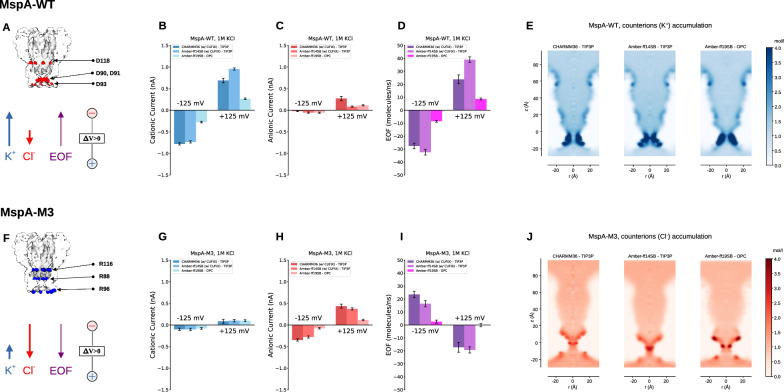
Effect of force field on MD simulation of flow through nanopores. **(A)** Sketch MspA-WT, with the most relevant acidic residues exposed towards the pore lumen colored in red. Simulations include membrane, water and ions (not shown) and are performed applying an electric field parallel to the pore corresponding to a voltage drop $\Delta V$ between the two reservoirs. The resulting cationic ($K^+$) and anionic ($Cl^-$) currents and EOF are in panels **(B-D)**. The bars correspond to the different force field. For MspA-WT, the cationic flow (blue) is larger than anionic one (red) as expected from the negative residues exposed towards the pore lumen. The negative residues also result in a counterions accumulation **(E)** that, coupled with the external electric field, give rise to an EOF. The bars in panel **(B-C)** and the three images in **(E)** correspond to different force fields. Panels **(F-J)** refer to MspA-M3 [159, 160], an anion selective nanopore. The figure is adapted from [161]

As a final biophysical tool to analyze the properties of nanopores, we mention MD in its classical all-atom version. In brief, each atom is modelled as a material point of mass *m* and charge *q* that interacts with other atoms via a conservative potential. The dynamics of the system is obtained by integrating Newton’s law of motion. In the last two decades, MD has been widely used in the analysis of nanopores. The contribution of MD in nanopore science can hardly be underestimated. MD allowed to investigate the mechanisms of transport of biomolecules through pores [162–168], to quantify pore selectivity and electroosmotic flow [35, 92, 93, 169–171] and, generally, to provide a mechanistic interpretation of the alteration of the ionic current signal observed in experiments [2, 50, 51, 163, 172–174]. MD is described in detail in several textbooks [175, 176]. Its specific application to transport through nanopores involves particular challenges, such as equilibrating the membrane protein and modeling the external forcing (which, in experiments, is usually applied far from the pore) within a triply periodic simulation box. These aspects require careful attention and consolidated protocols have been established in the last decades. Some of these protocols have been reviewed by us and other researchers [34, 52, 53, 177][?], we refer to mentioned references for a discussion. Here, we concentrate on two key issues that, in our opinion, have not yet been adequately reviewed.

#### Effect of the force field on transport properties

In MD, the potential energy that rules the interaction among atoms, usually indicated as the *force field* [178], is a key ingredient to accurately reproduce the phenomena of interest. Among the most widely used force fields are CHARMM [179] and Amber [180, 181] and, for each of them, updates are continuously released. Water and ions also need to be properly modelled. Over the past four decades, several water models have been developed to better approximate some physical and chemical properties of water [182, 183]. However, no classical water model is able to reproduce all the properties of water and, consequently, the choice of a model always implies some sort of systematic bias. For instance, the Transferable Intermolecular Potential with 3 Points (TIP3P) model [184], a variant of which is the standard water model for the CHARMM36 force field, has a viscosity that is $\simeq2.5$ times smaller than actual liquid water [185]. This implies that nanofluidic simulations with this model are not expected to be quantitatively accurate if intense liquid motion is present, as for instance, for electroosmotic flow in nanopores. A similar issue occurs for ions. Several corrections have been proposed to manage interactions among ions. Usually, these corrections amount to defining *ad-hoc* values for Lennard-Jones interactions (instead of relying solely on the Lorentz-Berthelot rule) for non-bonded ion-ion interactions [186, 187]. More recently, prosECCo, a novel force field based on CHARMM36, proposed a scaling of the atomic partial charges to improve the description of electrostatic interactions [188, 189]. In general, the main issue of non-equilibrium simulations aimed at calculating ion and water flow through nanopores is that force fields for macromolecules have not been originally developed considering non-equilibrium setting as a priority. Consequently, systematic errors on the prediction of flows have been largely tolerated by the community as, for the most cases, the actual value of the current is not the relevant information extracted from MD. For instance, when MD is used for studying electroosmotic flow (EOF), the focus is often on comparison between different mutants or on understanding of novel mechanisms of selectivity and EOF (such as the use of *sticky* ions [49], and the possibility to exploit an induced charge to obtain EOF in uncharged nanopores [190]). Consequently, a possible systematic disagreement in the quantitative prediction is not often going to affect the take-home message of the simulations.    

To test this, often implicit, assumption underlying MD application to nanopores, some of us recently performed a systematic comparison among three popular force fields. Specifically, the ionic current and EOF through cation and anion selective nanopores were studied using CHARMM36 [179], Amber ff14SB [180], (both with TIP3P water [184]), and Amber ff19SB [181] with OPC water [191]. The overall conclusion is that, while all force fields coherently reproduced the ion selectivity and the direction of EOF, quantitative differences were significant, especially for Amber-ff19SB/OPC, which yielded lower currents and EOF, (Fig. 5 where data for the MspA variants are reported). Although this difference may be somehow expected, since the viscosity of OPC is much larger than that for TIP3P, it cannot be fully explained by water viscosity and ion conductivity, as a simple linear rescaling of flux with transport coefficients is not sufficient to reconcile the results with different force fields. For instance, for MspA-WT, the EOF for the CHARMM36 and Amber-ff14SB vary even when using the same water model (TIP3P in both cases), (Fig. 5D). Additionally, MspA-M3 in Amber-ff19SB simulations is, essentially non-selective (anion and cation currents are similar, Fig. 5G-H, and the EOF values are negligible, unlike the other cases (Fig. 5I). Further analysis showed that the differences presumably originate from subtle variations in ion-protein or ion-water interactions, that are specific to each force field and affect the motion of the ions and their local distribution near the pore surface charges (Fig 5E and J). In general, these results support the use of MD for qualitative predictions in nanopores, but call for caution when extracting quantitative conclusions without validation, in particular when the surface charges of the nanopore are located in its constriction. Indeed, ion selectivity and EOF may be strongly affected by small variations in the arrangement of the charged residues exposed in the narrower region of the pore. A possible route to exclude that a specific result is an artefact of the force field, is to test different force fields/water model combinations in order to evaluate the robustness of the transport predictions. Alternatively, in some cases, experimental validation may be useful, although, as shown in the next section, for some of the quantities typically extracted from MD simulations, a direct comparison may be very challenging.

#### Connection with experiments

Nanofluidics is a blind field, *i.e.*, some of the most relevant quantities to characterize fluid and molecule transport in nanodevices are difficult or even impossible to measure experimentally. In the specific case of biological nanopores, the liquid flow, for instance, due to EOF, cannot be directly measured and also alternative approaches that rely on Onsager-like relations cannot be employed [192]. Similarly, direct access to the transport of a single ionic species is not possible. In essence, in the most common conditions, the only accessible quantity is the total ionic current flowing through the pore. This poses questions on the capability to experimentally validate MD findings and, more in general, on how MD and experiments can be used to understand the flow through nanopores. To try to understand how MD and experiments can be connected and/or compared, it is instrumental to recall on the one hand which are the typical experimental set-ups used to infer EOF and selectivity and, on the other hand, the main criticalities of MD simulation aimed at studying the flow through nanopores.

A widely employed approach to estimate the differential permittivity of a nanopore for different ionic species is to measure the reversal potential. The experiment is relatively straightforward: the two reservoirs are at different salt concentrations and a voltage $\Delta V$ is applied between them. This allows to collect an IV curve. If the pore is selective for cations (or anions), the different diffusive fluxes of anions and cations will result in an electric current even at $\Delta V = 0$ and the current will be null for a voltage $\Delta V$ that is commonly indicated as the reversal potential $V_r$. The sign of $V_r$ depends on the anion/cation selectivity of the pore. In the simple case where only two ionic species are present in the electrolyte solution, the permeability ratio $P_+/P_-$ (the ratio between anion and cation currents) can be obtained from $V_r$ using the Goldman-Hodgkin-Katz (GHK) model [193, 194]. GHK is a simplified theoretical model of the ion transport under the action of an electrochemical gradient and, consequently, it relies on several assumptions. It is worth noting that there are other theoretical descriptions that may be used to link the permeability ratio to the reversal potential, as, for instance, a Nernst-like approach often used in the field of salinity gradient energy harvesting [19, 195]. The comparison between the two approaches and the limitations of GHK are out of the aim of the present review and we refer the interested readers to [196, 197]. The main points we would like to stress here are that i) GHK provides an approximated expression of the permeability ratio and ii) this estimation may also slightly depend on the specific values of the salt concentration as shown in [198]. Concerning EOF, instead, there is no rigorous way to estimate it from the permeability ratio, although a simplified purely kinematic theory was proposed more than two decades ago [89]. For a more detailed discussion on the limits of the EOF model proposed in [89] (and widely used in the nanopore literature [5, 48, 93, 199, 200]) we refer to recent work of some of the authors [192]. In summary, experiments on narrow biological nanopores allow an estimation of the permeability ratio, although with some caveats, but they do not allow an estimation of EOF.

MD provides a complementary perspective on the transport. Indeed, from MD it is possible to directly quantify the fluxes of any ionic species and the flux of water. In principle, this should allow a direct connection between experiments and MD. Indeed, both approaches provide a measurement on the permeability ratio $P_+/P_-$. Unfortunately, this quantitative comparison is often not robust or not possible. First, as already pointed out, $P_+/P_-$ estimated from experiments depends on the concentration difference between the two reservoirs and it is systematically and intrinsically affected by the approximations of GHK model (or of alternative models) [196, 197]. Second, the estimation of permeability ratio from MD is largely affected by statistical errors. Simulating the systems at larger voltages to increase the signal-to-noise ratio will not completely solve the issue as often the IV curves of nanopores show strong non-linearities even at small voltages (100–200 mV). A possibility to overcome this issue would be to perform a simulation in a condition similar to the experiments, *i.e.* with a concentration difference between the reservoirs. However, this poses two connected issues, one practical and one theoretical. On the practical side, MD simulations need to be triperiodic so that electrostatic interaction can be efficiently handled with Ewald summation and PME algorithm. This poses a challenge on how to impose a concentration gradient, although some strategies have been proposed [201, 202]. On the theoretical side, periodicity implies that the simulated system is not a single nanopore but an infinite 2D array of nanopores. For diffusive fluxes the passage from a single nanopore to an array drastically changes the topology of the transport exacerbating concentration polarization effects. In a nutshell, while for a single nanopore the diffusive flux funnels into the pore and, consequently, the size of the reservoir does not affect the diffusive current 2 this is not the case for an infinite array of nanopores [19, 203–205]. In summary, in our view, there are no clear ways to quantitatively compare selectivity and EOF from MD and experiments. Despite this limitation, that is shared with several other areas of molecular biology and nanotechnology, MD remains an extremely powerful tool to preliminary explore strategies to be implemented in experiments, to propose and corroborate mechanistic interpretations of the experimental data and to support the design of nanopore based devices.

## De novo design of nanopores

**Fig. 6:**
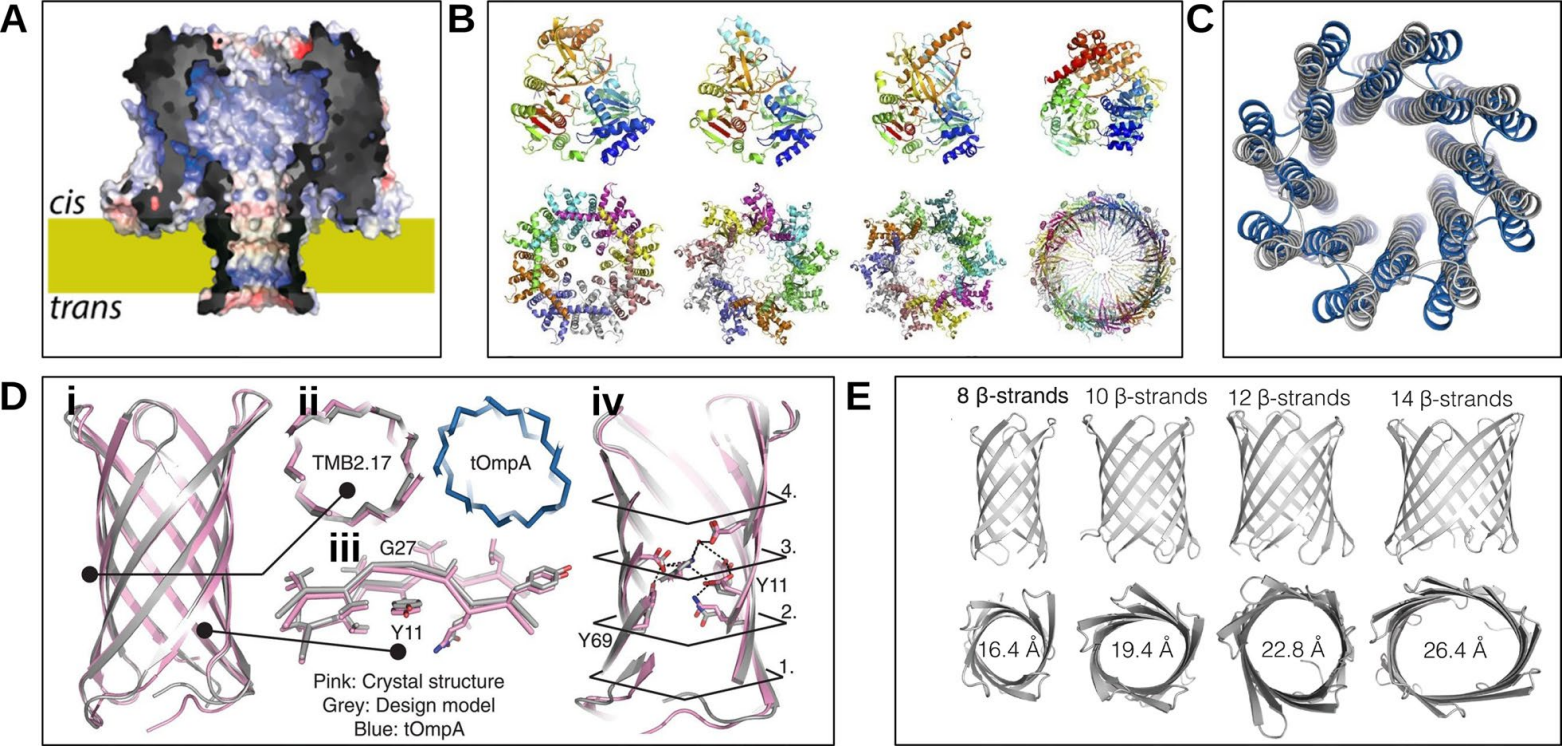
Representative examples of *de novo* nanopore structure predictions. **(A)** Cut-through view of the CytK nanopore predicted by homology modeling using SWISS-MODEL server version, shown as surface potential maps highlighting positively (blue) and negatively (red) charged regions. Adapted from [211]. **(B)** Schematic diagram of AlphaFold3 predicted structure of candidate helicases (top) and pore-forming proteins (bottom) for CycloneSEQ. Adapted from [213]. **(C)** Superposition of the backbones of the crystal structure (blue) and the design model predicted by the Rosetta ’fold-and-dock’ method (grey) of WSHC8. The $\text {C}_{\alpha }$ RMSD between the crystal structure and the design model is 2.51 Å (octamer) and 0.97 Å (monomers). According to the authors, the larger deviation for the octamer is caused by the slight tilting of the hairpin monomers along the superhelical axis of the complex. Adapted from [214]. **(D)(i)** Crystal structure of TMB2.17 (pink) determined in DPC detergent, superimposed on the Rosetta design model (grey), and compared with the crystal structure of the naturally occurring tOmpA (blue; PDB ID: 1BXW [215]). **(ii)** Comparison of transverse $\beta$-barrel cross-section geometries. **(iii)** Full backbone superposition. **(iv)** Superposition of the $\beta$ strands around a mortise–tenon motif, showing the extended backbone conformation of the glycine kink (G27) and the rotamer of the tyrosine involved in the aromatic rescue interaction (Y11), which are nearly identical in crystal structure and design model. Adapted from [46]. **(E)** Transmembrane $\beta$-barrels with different diameters predicted by Rosetta BlueprintBDR. Barrel diameter can be controlled through the number of $\beta$-strands

*De novo* protein design is a rapidly evolving field with great potential for technological innovation. Rather than modifying natural proteins, these approaches aim to create entirely new macromolecules tailored to specific tasks, sometimes beyond the boundaries of biology. Several reviews provide comprehensive overviews of this area [206–209], to which we refer the reader for a broader perspective. Representative examples of application to nanopores are summarized below to illustrate the diversity of current approaches, rather than to provide an exhaustive or unified view, a choice we made given the rapid evolution of this field (Fig. 6).

### Homology modelling

The earliest approaches to structural prediction were based on homology modelling. Starting from a correct alignment between a target sequence and homologous proteins with known structures (templates), a three-dimensional model could be constructed. This strategy is simple and effective when templates are available, but it cannot generate entirely novel folds. Only a few examples of nanopores modelled by homology can be found in the literature. For instance, the $\beta$-barrel nanopore Cytotoxin K (CytK) was reconstructed using SWISS-MODEL [78] employing $\alpha$-hemolysin [210] as template [211] (Fig. 6A). A more complex strategy was used for the two-component nanopore pleurotolysin A/B (PlyAB) [57], where MODELLER [77] was employed to construct and refine an all-atom model starting from a low resolution cryo-EM map and crystal structures of the PlyA and PlyB monomers [212].

### Alphafold

In the last few years, artificial intelligence has revolutionized the field of protein structure prediction. Among the most prominent tools is AlphaFold [216–218], which is based on deep learning techniques trained on structural data. AlphaFold can predict three-dimensional structures without requiring explicit templates. This approach has transformed structural prediction, achieving high accuracy even in the absence of apparent homology [219]. AlphaFold2 is particularly effective in generating high-resolution models for individual protein chains. However, its original version was limited to monomeric structures, while the prediction of oligomers and protein complexes requires AlphaFold-Multimer [220]. The most recent version, AlphaFold3 [218], has been further improved to model protein–nucleic acid and protein–ligand complexes.

In the context of nanopore research, Hermosilla et al. [221] have shown that AlphaFold2, (through ColabFold [222]), can reliably predict the structure of newly designed transmembrane $\beta$-barrels, even in the absence of multiple sequence alignments and templates. A recent preprint [213] applied AlphaFold3 to predict unsolved structures of porins, revealing possible oligomeric states compatible with membrane insertion, with internal lumens large enough to accommodate DNA translocation for nanopore-based sequencing, Fig. 6B.

**Fig. 7:**
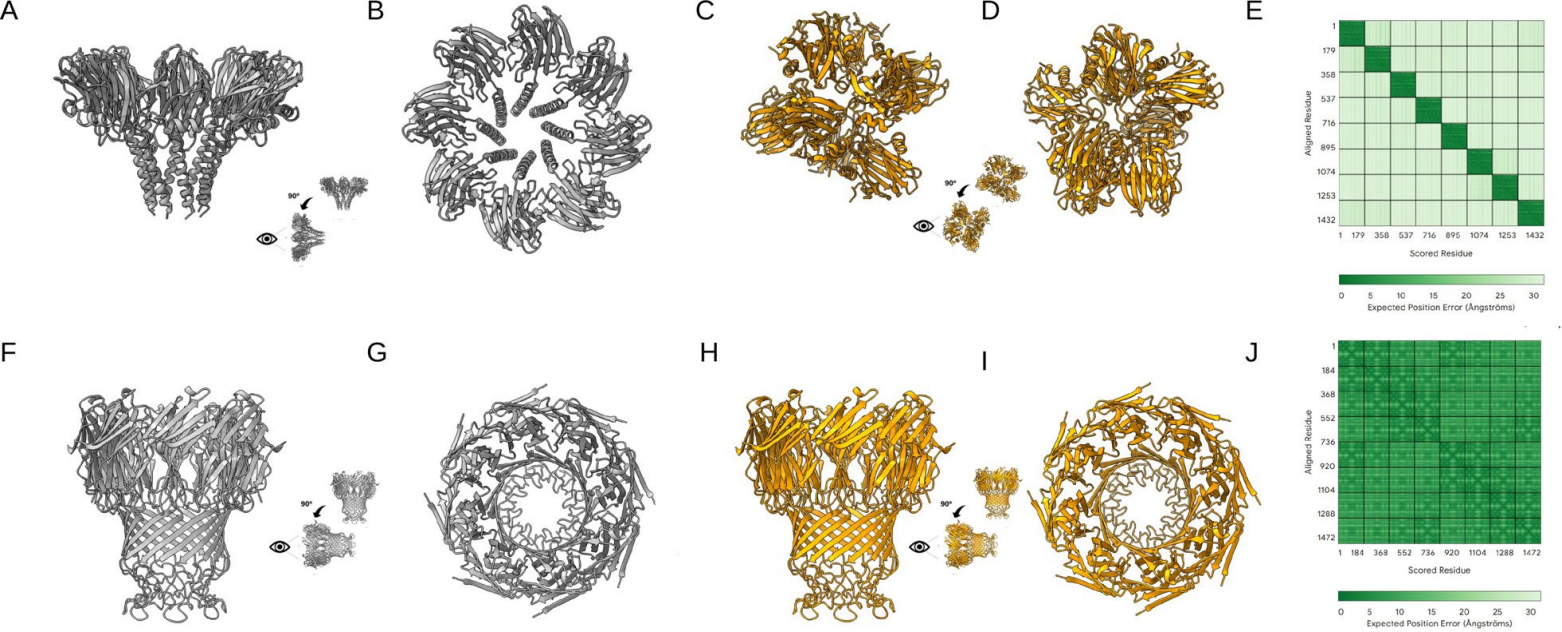
Examples of AlphaFold3 predictions for nanopores. Structures were predicted using AlphaFold3 (Custom Template with Auto selection up to February 3, 2025) for two nanopores: FraC and MspA. In both cases, eight copies were selected in the AlphaFold3 server interface. **(A, B)** Side and top views of the crystal structure of FraC (PDB: 4TSY [84]). **(C, D)** AlphaFold3 predicted structure for FraC. **(E)** PAE (predicted aligned error, a default output of the AlphaFold3 Server) map: Higher PAE values (light green) correspond to larger predicted error and therefore lower confidence. **(F-G)** Crystal structure of MspA (PDB: 1UUN [223]). **(H-I)** AlphaFold3 prediction for MspA. In this case, the PAE map **(J)** shows lower values (dark green) compared to the FraC case

As examples of the capability of AlphaFold3 to reliably predict nanopore conformations, we present here two cases: FraC and MspA nanopores, both of which have crystallographic structures available in the PDB database [38]. For FraC, the AlphaFold3-predicted structure deviates from the experimental model (PDB: 4TSY [84]), (Fig. 7A-E). In contrast, for MspA, the predicted structure closely matches the experimental one, (Fig. 7F-J). Supplementary Figure S6, reports an additional example where the mutation of a single residue alters the prediction. These occurrences highlight the need for caution in the uncritical use of AlphaFold3. Importantly, AlphaFold provides confidence metrics, such as the predicted Local Distance Difference Test (pLDDT) and the Predicted Aligned Error (PAE), see Fig. 7E,J, which, when properly interpreted, can help assess the reliability of a predicted model.

As a final note, we report that, during the review process, a preprint [224] presented NanoporeDB, an open-access structural database containg thousands of multimeric models for nanopore. However, at the time of the writing, the website of NanoporeDB is not public.

### Rosetta and RoseTTAFold

The Rosetta suite [225, 226] (that includes a wide range of tools for modelling and analysing macromolecules) and its deep learning-based extension RoseTTAFold [227] have been used for *de novo* design of transmembrane pores. Xu et al. [214] applied a combination of Rosetta tools to design a *de novo* helical pore, producing models that closely matched the experimental crystal structure ($C_\alpha$ RMSD $\sim 2.5$Å), Fig. 6C. Rosetta has also been employed for *de novo* design of transmembrane $\beta$-barrels: Vorobieva et al. [46] reported new eight-stranded $\beta$-barrel pores, whose Rosetta models closely matched experimentally resolved crystal structures, Fig. 6D. Berhanu et al. [136] further extended this methodology, providing a general approach to design monomeric transmembrane $\beta$-barrel nanopores with different diameters and shapes, Fig. 6E. RoseTTAFold was employed for *de novo* design of $\beta$-barrel nanopores as shown by Kim et al. [228]. In their workflow, cylindrical backbones of varying radii were first generated, and an improved version of RoseTTAFold was then used to complete the structures. This approach potentially enables parametric control over pore shape and allows the automatic incorporation of structural irregularities required for correct folding [228]. Finally, we mention another application of Rosetta tools, not directly related to *de novo* design, concerns the exploration of different oligomerization states. For example, Huang et al.[229] reported alternative oligomeric arrangements (from pentamer to nonamer) of the octameric FraC pore, constructed by recombining monomers via the symmetrical docking function of Rosetta [230].

### Other tools

In addition to the tools mentioned in the previous paragraphs, with the advancement of artificial intelligence, numerous tools for protein design and modeling have emerged. Tracking and reviewing all of them is beyond the scope of this review. In this section, we highlight a few that, in our opinion, may have application to nanopore design.

Boltz-2 [231] and Chai-1 [232] are two novel tools for structure prediction and ligand interaction modeling within a single framework. To the best of our knowledge, no peer-reviewed applications of Boltz-2 and Chai-1 to biological nanopores have been published to date. Another promising tool is ProteinMPNN [233] which, given a suitable protein backbone, generates compatible amino acid sequences. Dolorfino et al. [234] tested ProteinMPNN on transmembrane $\beta$-barrels and demonstrated that the tool can recover complex features of natural sequences, generating candidates that are nearly native and pass state-of-the-art *in silico* filters [234]. These findings suggest ProteinMPNN could be used for *de novo* design of nanopores for single-molecule sensing and sequencing applications. This method allows fast exploration of sequence space and can utilize existing structures as input (be it mono- or multimeric). However, the quality of the generated sequences strongly depends on the accuracy of the input backbone. In particular, for $\beta$-barrels, limitations have been reported when the backbone contains non-native geometries or features [234].

A rigorous quantitative comparison of AI-based generative methods, AlphaFold-based structure prediction, and classical homology or Rosetta-based approaches would require standardized datasets and evaluation protocols and is therefore beyond the scope of this study. Such systematic assessments are instead provided by community-wide benchmarking efforts such as the Critical Assessment of Protein Structure Prediction (CASP; https://predictioncenter.org/), including the most recent CASP16 round [235].

## Conclusion

In this review, we have discussed some computational tools that can support the design of biological nanopores. We have not attempted to cover all structural bioinformatics methods, but focused on those already applied to nanopores, such as Poisson–Boltzmann calculations, and on selected approaches that, although not yet tested in this context, may provide useful insights, for instance, the prediction of pore flexibility and of accessibility to the solvent. For several tools (such as single-point mutations, APBS and estimation of the protonation states) we provided scripts and protocols in the Supplementary Information so that the readers can apply and adapt them to their systems. In the last section, we also attempted to explore the field of *de novo* protein design. However, the field is rapidly evolving, with new tools continuously emerging. Therefore, this overview should be considered a starting point rather than an exhaustive survey.

Our view is that the full potential of bioinformatic tools has not yet been displayed in the nanopore field. Bioinformatics analyses can be especially valuable in the early stages of nanopore design, when experimental structures are missing or molecular dynamics simulations are not feasible on a large scale. At the same time, different tools may lead to divergent predictions, as we have shown, for example, with single-point mutations and protonation state estimation, and therefore results should be interpreted with care and supported by internal consistency checks, such as the comparison of multiple tools or parameter settings. These predictions can then be integrated with atomistic simulations to refine the models (ideally using multiple force fields) and guide experimental validation, making the combined approach a powerful strategy for the engineering of novel nanopores.

## Supplementary Information

Supplementary file 1: The included supplementary materials provide practical resources to support and extend the analyses presented in this work. They include a GitHub repository, annotated scripts, and commandline guides required to reproduce and further explore the discussed computational procedures. Together, these resources are intended to serve as a hands-on companion to the review, enabling readers to apply, adapt, and expand the presented approaches for their own nanopore-related investigations.

## Data Availability

Data supporting the findings of this study are available within the paper and its Supplementary Information files. Should any raw data files be needed in another format they are available from the corresponding author upon reasonable request.

## Footnotes

1 For details, see the Official Chimera User’s Guide, in the section Basic Commands at Chapter 7 Commands -swapaa

2 More precisely, for size of the reservoirs that goes to infinity the ionic currents converge to a finite, non-zero, value
