# Supplementary material for: Bioinformatic and computational biophysics tools for nanopore engineering: a review from standard approaches to machine learning advancements
Source: J Nanobiotechnology. 2026 Mar 5;24:343. doi: 10.1186/s12951-026-04225-4 (PMC13072578; doi:10.1186/s12951-026-04225-4)
Supplement: Supplementary file 1 — The included supplementary materials provide practical resources to support and extend the analyses presented in this work. They include a GitHub repository, annotated scripts, and commandline guides required to reproduce and further explore the discussed computational procedures. Together, these resources are intended to serve as a hands-on companion to the review, enabling readers to apply, adapt, and expand the presented approaches for their own nanopore-related investigations. [file 12951_2026_4225_MOESM2_ESM.pdf]

Supplementary Information for: Bioinformatic and computational biophysics tools for nanopore engineering: a review from standard approaches to machine learning advancements

Marco Reccia[a], Francesco Quilli[b], Kherim Willems[c], Blasco Morozzo della Rocca[d], Domenico Raimondo[b], Mauro Chinappi[a,*]

[a] *Department of Industrial Engineering, University of Rome Tor Vergata, Italy*
[b] *Department of Molecular Medicine, Laboratory Affiliated to Istituto Pasteur-Fondazione Cenci Bolognetti, Sapienza University of Rome*
[c] *imec, Belgium and*
[d] *Department of Biology, University of Rome Tor Vergata, Italy*

Corresponding author: mauro.chinappi@uniroma2.it;
Contributing authors: marco.reccia@alumni.uniroma2.eu; francesco.quilli@uniroma1.it; kherim.willems@imec.be; blasco.morozzo.della.rocca@uniroma2.it; domenico.raimondo@uniroma1.it.

Supplementary Note S1: Mutagenesis Protocols in PyMOL, ChimeraX, and VMD p. S-2
Supplementary Note S2: PROPKA3 and H++ Protocols for pK_a Predictions p. S-7
Supplementary Note S3: Electrostatic potential with APBS p. S-9

Supplementary Figure S1: Mutations comparison in MspA p. S-13
Supplementary Figure S2: Effect of ionic concentration on pK_a predictions p. S-14
Supplementary Figure S3: Effects of D118R modeling on the electrostatic potential of MspA p. S-15
Supplementary Figure S4: Sensitivity of the electrostatic potential to APBS parameters. p. S-16
Supplementary Figure S5: Volume of the nanopore lumen p. S-17
Supplementary Figure S6: Additional data on MspA and FraC modelling with AlphaFold3 p. S-19

Supplementary Table S1: Overview on rotamer selection strategies in modeling tools p. S-20
Supplementary Table S2: PROPKA3-predicted pKa values for α-hemolysin p. S-21
Supplementary Table S3: H++-predicted pKa values for α-hemolysin p. S-24
Supplementary Table S4: PROPKA3 and H++ comparison of pKa values for α-hemolysin p. S-27
Supplementary Table S5: PROPKA3-predicted pKa values for FraC p. S-30
Supplementary Table S6: H++-predicted pKa values for FraC p. S-32
Supplementary Table S7: PROPKA3 and H++ comparison of pKa values for FraC p. S-34

Supplementary references: p. S-36

Preliminary note: The Supplementary Notes S1, S2 and S3 contain some scripts and protocols used in the manuscript. We ran those scripts on GNU/Linux operating systems, although, to the best of our knowledge, the software used (VMD [S1], PyMOL [S2], UCSF ChimeraX [S3, S4]) are available also on other operating systems. The examples and scripts assume a Unix-like command-line environment (bash) and the availability of basic shell commands (e.g., file copying, globbing, and output redirection). For non-Linux users, we suggest installing a virtual machine with GNU/Linux. Alternatively, they can install the individual software and a bash emulator. Concerning the required software dependencies, we refer to the documentation of the individual software used.

SUPPLEMENTARY NOTE S1: MUTAGENESIS PROTOCOLS IN PYMOL, CHIMERAX, AND VMD

The following protocols describe the procedures employed for introducing point mutations across all chains of the target nanopore, present in 2.1 and Fig. 2 of article, using PyMOL 3.1 [S2], UCSF ChimeraX [S3, S4], and VMD 1.9.4a53 [S1]. Since our VMD script requires as an input a structure in which each chain is complete (and not fragmented in segments separated by missing residues), the structure of CsgG is obtained by AlphaFold3 [S5] (server version). Specifically, to obtain this structure we used the fasta sequence of PDB ID:4UV3 [S6] in RCSB PDB web server https://www.rcsb.org/structure/4UV3. We then pasted the sequence in the AlphaFold and chose 9 as number of monomers in the variable "Copies". We selected the first model (model number 0) proposed by AlphaFold3 that, according to AlphaFold3 guidelines, is the best. The AlphaFold3 output in .cif (csgg.cif) format was then converted to .pdb format using the software Gemmi 0.7.4 with the command `gemmi convert csgg.cif csgg.pdb`. (it's also possible to use the web server version also https://project-gemmi.github.io/wasm/convert/cif2pdb.html). The resulting AlphaFold3 model shows a backbone RMSD of 0.5 Å relative to the experimentally resolved PDB ID:4UV3 structure, thus maintaining a close structural agreement with the native configuration. While this approach proved effective in our evaluations, in most cases an homology modeling approach would represent a safer and more controlled choice, as it allows one to completely preserve experimentally resolved regions of the protein while modeling only the missing or unresolved parts. Structure of MspA (PDB ID:1UUN [S7]) used for mutagenesis was retrieved directly from the OPM (Orientations of Proteins in Membranes) [S8], database to ensure membrane-aligned orientation. In the following, we assume that the scripts are run in a Unix-like terminal.

PyMOL 3.1 (Mutagenesis Wizard, Scripted Automation)

The mutation was introduced using PyMOL's built-in *Mutagenesis Wizard*, scripted in Python for batch execution. To run the script, save the code below as `mutate.py`.

```
# Load structure and activate mutagenesis wizard
load input.pdb, mol
wizard mutagenesis
refresh_wizard

# Apply mutation to all chains at residue 51
python
from pymol import cmd

target_resi = 51              # Residue number
target_aa = "ARG"             # Target amino acid
structure = "mol"

cmd.get_wizard().set_mode(target_aa)

for chain in cmd.get_chains(structure):
    selection = f"/{structure}//{chain}/{target_resi}"
    cmd.get_wizard().do_select(selection)
    cmd.get_wizard().apply()

cmd.set_wizard()
cmd.save("output.pdb", structure)
```

```
cmd.quit()
python end
```

Then, launch PyMOL from terminal with the command

```
pymol -cq mutate.py
```

Rotamers were selected by default based on the highest statistical probability from the Dunbrack library [S9]. This script loads a PDB structure (`input.pdb`) and introduces a point mutation at the residue `target_resi` (51 in the script) across all chains, replacing the original residue with a user-defined target amino acid `target_aa` (ARG in the script). The mutated structure is saved as `output.pdb`.

ChimeraX (Command-Line Rotamer Substitution)

The same mutation can be obtained in ChimeraX using the `swapaa` command, specifying each chain manually. That is, the command `swapaa /A:51 ARG` replaces the residue at position 51 in chain A with an arginine. To run the script, I save the following commands into a script file (e.g., `mutate.cxc`). Then open ChimeraX GUI, go to the menu "File", click on "Open" and select the script.

```
open input.pdb
swapaa  /A:51 ARG
swapaa  /B:51 ARG
swapaa  /C:51 ARG
swapaa  /D:51 ARG
swapaa  /E:51 ARG
swapaa  /F:51 ARG
swapaa  /G:51 ARG
swapaa  /H:51 ARG
swapaa  /I:51 ARG
save output.pdb format pdb
exit
```

Rotamer selection in ChimeraX was performed using a hierarchical multi-criteria procedure (*dchp*), where sidechains were evaluated first by their fit to the electron density map (when available), followed by minimization of steric clashes with surrounding atoms (excluding interactions with alternative rotamers and the original sidechain), maximization of hydrogen bond formation, and finally by their statistical prevalence in the rotamer library (e.g., Dunbrack), which accounts for the backbone ϕ and ψ angles. If no electron map was available and density fit was not explicitly requested, the first criterion was skipped automatically.

VMD 1.9.4a53 (Tcl Scripting with PSFGEN and Mutator Plugin)

VMD was used to perform residue mutation via Tcl scripting. The procedure requires to upload CHARMM36 topology files. In the following example, we expect that a directory `toppar` containing the appropriate topology files is present in the same directory of the script. Moreover, a directory `Temp` to execute the mutation script, save it as `mutate.tcl`.

```
package require autopsf
package require psfgen
resetpsf

set pdb   input.pdb
set resid  51
set newres ARG
set outbase output

set topo {
  toppar/top_all36_prot.rtf
}


mol new $pdb
autopsf -mol top -top $topo -protein -regen
file mkdir Temp
file copy -force [glob *_autopsf.psf] Temp/prot.psf
file copy -force [glob *_autopsf.pdb] Temp/prot.pdb
file delete {*}[glob *_autopsf*]
mol delete all

mol new Temp/prot.psf
mol addfile Temp/prot.pdb waitfor all
set segs [lsort -unique [[atomselect top protein] get segname]]
foreach seg $segs {
  set sel [atomselect top "segname $seg"]
  $sel writepdb Temp/seg_${seg}.pdb
  $sel writepsf Temp/seg_${seg}.psf
  $sel delete
}
mol delete all

file mkdir Temp/out_segs
foreach seg $segs {
  puts "Processing chain $seg"
  resetpsf
  segment $seg {
    pdb Temp/seg_${seg}.pdb
    mutate $resid $newres
  }
  coordpdb Temp/seg_${seg}.pdb $seg
  guesscoord
  regenerate angles dihedrals
```

```
  writepsf Temp/out_segs/${seg}.psf
  writepdb Temp/out_segs/${seg}.pdb
}

resetpsf
foreach psf [lsort [glob Temp/out_segs/*.psf]] {
  readpsf $psf
}
foreach pdb [lsort [glob Temp/out_segs/*.pdb]] {
  coordpdb $pdb
}

writepsf ${outbase}.psf
writepdb ${outbase}.pdb
exit
```

Then, launch the script from terminal with

```
vmd -dispdev text -e mutate.tcl
```

To the best of our knowledge, VMD does not include automated rotamer selection based on energetics or statistical probability. Rotamers are directly taken from the topology of the force field used, in this case CHARMM36.

SUPPLEMENTARY NOTE S2: PROPKA3 AND H++ PROTOCOLS FOR pK_a PREDICTIONS.

To assess the variability between different computational tools in predicting residue protonation, we compared the results of two widely used pK_a prediction methods: PROPKA3 [S10] and H++ [S11].

For PROPKA3, we used the local command-line software, version 3.5.1, executed via:

```
propka3 input.pdb
```

where `input.pdb` is the structure file in PDB format.

For H++, we employed the web server (version 4.0) using the following physical parameters:

- Salinity: 0.15 M
- Internal dielectric constant: 10
- External dielectric constant: 80
- Reference pH: 7.0

For α-hemolysin, we used the crystallographic structure from the RCSB Protein Data Bank (PDB ID: 7AHL [S12]). For FraC, we used the membrane-aligned OPM structure (PDB ID: 4TSY [S13]). Since OPM [S8] files include artificial `HETATM` records used to orient the transmembrane domain, these were removed using VMD. Furthermore, the H++ server requires explicit `TER` records to indicate the end of protein chains, which are often missing from OPM files. To address this, we developed a custom Python script to append `TER` lines at chain boundaries.

```python
def process_pdb(input_path: str, output_path: str):
    with open(input_path, 'r') as f:
        lines = f.readlines()

    output_lines = []
    atom_serial = 1
    prev_chain = None
    last_atom_line = None

    for i, line in enumerate(lines):
        if line.startswith(('ATOM', 'HETATM')):
            chain_id = line[21]
            if prev_chain is not None and chain_id != prev_chain:
                # Insert a TER line before the new chain starts
                ter_line = create_ter_line(atom_serial, last_atom_line)
                output_lines.append(ter_line)
                atom_serial += 1

            # Renumber atom serials and write ATOM line
            new_line = update_atom_line(line, atom_serial)
            output_lines.append(new_line)
            atom_serial += 1
            last_atom_line = line
            prev_chain = chain_id
        else:
```

```
            continue  # Skip lines that are not ATOM/HETATM

    # Insert final TER line
    if last_atom_line:
        ter_line = create_ter_line(atom_serial, last_atom_line)
        output_lines.append(ter_line)

    with open(output_path, 'w') as f:
        f.writelines(output_lines)

def update_atom_line(line: str, new_serial: int) -> str:
    return f"{line[:6]}{new_serial:5d}{line[11:]}"

def create_ter_line(serial: int, atom_line: str) -> str:
    res_name = atom_line[17:20]
    chain_id = atom_line[21]
    res_seq = atom_line[22:26]
    return f"TER   {serial:5d}      {res_name} {chain_id}{res_seq}

if __name__ == "__main__":
    import sys
    if len(sys.argv) != 3:
        print("Usage: python renumber_pdb.py input.pdb output.pdb")
    else:
        process_pdb(sys.argv[1], sys.argv[2])
```

This script processes an input PDB file and inserts appropriate `TER` records between chains, renumbering atoms consecutively. Although the script can process PDB files containing `HETATM` entries, both PROPKA and H++ documentation recommend removing these before analysis to avoid inaccuracies in pK_a predictions.

SUPPLEMENTARY NOTE S3: ELECTROSTATIC POTENTIAL CALCULATIONS WITH APBS.

With reference to the considerations on the electrostatic potential shown in Figure 4 of the main text, here we explain the computational procedure used to obtain these results. All electrostatic calculations were performed with APBS [S14, S15] using a Bash workflow composed of two separate scripts. The protocol begins by converting the PDB structure into a PQR file with charge and radius assignments using PDB2PQR. Next, coefficient maps for dielectric (“`diel`”), ionic accessibility (“`kappa`”), and atomic charge (“`charge`”) functions are generated through an initial “dummy” APBS run (“`mg-dummy`”). A low-dielectric membrane slab is then inserted into these maps using the utility `draw_membrane2` included with the APBS source code. Finally, a second APBS calculation (`mg-manual`) reads the modified maps and computes the electrostatic potential in two “focusing” steps: a coarse grid calculation with approximate boundary conditions (BCs) followed by a fine grid that used the coarse results as BCs. In the following section, we provide a simple description of every command and argument used, to allow the entire calculation to be reproduced manually after installing APBS and PDB2PQR. The automated Bash pipeline, available in the GitHub repository `https://github.com/willemsk/nanopore-simulation-tools`, offers the same procedure in a streamlined form (e.g., to automate the calculation of many nanopore variants or electrolyte conditions in parallel).

Stage 0: Preparation of the nanopore PDB file

To enable the computational workflow with APBS, the nanopore PDB-file should be modified to meet the following requirements:

- The nanopore channel axis should be aligned along the z-axis, and centered around the $(x, y, z) = (0, 0, 0)$ coordinates, with the nanopore axis coinciding with the z-axis and the center of the bilayer at $z = 0$ The latter is not strictly needed, but it simplifies the calculation settings later on.
- The nanopore should explicitly contain all structurally and electrostatically relevant residues. In some cases (e.g., if entire residues or flexible loops are missing), this means using molecular modelling tools such as homology modelling and molecular dynamics simulation to (re)build the most likely conformation of those missing atoms.
- Once a suitable wild-type structure is obtained, it can then be modified to any desired variant (i.e., point mutations applied within reason).

Note that for most nanopores, a good starting point for creating a structure that meets the alignment requirements is available from the orientations of proteins in membranes (OPM) database [S8, S16]. These structure files also contain dummy atoms that indicate the (likely) top and bottom positions of thelipid bilayer. Hence, their z-coordinates can be used to determine the z-offset and membrane thickness, which are inputs for the `draw_membrane2` program. Finally, as most nanopores have a water-filled channel, this region must be removed from the dielectric slab created by the `draw_membrane2` program. A good rule of thumb is to find the radii of the water-filled channel up until the middle of the protein “wall” at the top and bottom leaflets of the bilayer. This can be done visually using PyMOL. After loading the membrane-oriented nanopore structure (*e.g.*, MspA, PDB ID: 1UUN [S17]) from the OPM database, one can inspect the DUM residues marking the bilayer boundaries and measure the channel’s lateral extent in the x–y plane. The following PyMOL commands exemplify this process, showing how to isolate the bilayer, identify its top and

bottom halves, and determine the approximate radial coordinates corresponding to the upper and lower cutouts used in draw_membrane2. For example: after downloading the oriented PDB file for MspA (1uun [S17]) from the OPM database, one can visually determine the top and bottom radii in PyMOL using the following commands:

```
delete all
load structure.pdb

select nanopore, (pol)

extract bilayer, resname DUM
select bilayer_top, bilayer and z>0
select bilayer_bot, bilayer and z<0

remove (not bilayer and solvent)

print(cmd.get_coords("bilayer_top", 1)[0][2])
print(cmd.get_coords("bilayer_bot", 1)[0][2])

select nanopore_slice, (nanopore and z> -20.35 and z< 20.35)

hide everything
show spheres, nanopore_slice
show dots, bilayer

# Store the radial coordinate of each atom in the B-factor
alter_state 0, all, b=(x**2 + y**2)**0.5

select bilayer_top_channel, (bilayer_top and b<23.2)
select bilayer_bot_channel, (bilayer_bot and b<17.5)

show spheres, bilayer_top_channel
show spheres, bilayer_bot_channel
```

In these commands, the arguments define the geometric and physical properties of the inserted membrane. The first value specifies the z-coordinate of the lower leaflet of the bilayer (-18 Å), effectively setting the vertical position of the membrane in the simulation box. The second value indicates the bilayer thickness (36 Å), defining the width of the low-dielectric slab that represents the hydrophobic core of the membrane. The third argument sets the dielectric constant of the protein within the membrane region (10.0). The fourth value corresponds to the transmembrane potential (0.0 V), which is set to zero in this example. The fifth argument defines the bulk salt concentration (0.15 M), representing the ionic environment. Finally, the last two values indicate the radii of the cone-shaped cutouts in the membrane that prevent the dielectric slab from intruding into the pore lumen, upper (23.2 Å) and lower (17.5 Å), thus keeping the channel's interior solvent-accessible. These parameters collectively ensure that the resulting coefficient maps accurately reflect both the presence of the membrane and the accessible solvent regions inside the protein pore.

Stage 1: PDB to PQR (PDB2PQR)

Once the structure file is fully prepared, it can be converted to PQR format with PDB2PQR so

that APBS receives explicit per-atom charges and radii. This way protonation states can be set to a chosen pH. An example bash command used in this work is

```
pdb2pqr30 \
  --log-level INFO \
  --userff=../params/mycharmm_spl2.dat \
  --usernames=../params/mycharmm.names \
  --whitespace \
  --with-ph=7.0 \
  ../output/pore-mutants/pdb/mutant1_oriented.pdb \
  ../output/pore-mutants/pqr/mutant1_oriented_pH7.0.pqr \
  > ../output/pore-mutants/pqr/mutant1_oriented_pH7.0.log 2>&1
```

In this command, `--log-level INFO` captures and redirects diagnostic information from the conversion to a .log file, the option `--with-ph=7.0` instructs PDB2PQR to assign protonation states consistent with physiological pH, while the arguments `--userff` and `--usernames` allow the use of a custom CHARMM-style force field and atom-name mapping, which we provide in the repository but can be replaced with any compatible files or with the standard, built-in forcefields using the `--ff` flag. The `--whitespace` flag ensures that the resulting PQR file remains compatible with older APBS parsers, and the output PQR file generated becomes the direct input for APBS (referred to as TM.pqr in later examples). Note that more options are available to customize the PDB2PQR behavior, and we encourage the reader to look up the official documentation. The directory structure shown above is only an example for clarity: users are free to organize their working directories and subdirectories in whichever way best suits their project. This step produces identical results whether executed through the automated wrapper or manually, with the wrapper being extremely helpful to automate the process, making it easier to loop over multiple structures and conditions.

Stage 2 - Build coefficient maps with the "dummy" APBS run

After producing the PQR file, the pipeline performs a "dummy" APBS run using the `mg-dummy` multigrid directive in the provided `apbs_dummy.in` file. The mg-dummy directive in APBS is a special multigrid setup that constructs the numerical grids and coefficient maps required for solving the Poisson-Boltzmann equation but does not perform the actual electrostatic calculation. Instead, it writes to disk the dielectric maps (dielx, diely, dielz), ion-accessibility maps (kappa), and charge distributions (charge). These are generated at two focus levels: the large focus, which corresponds to a coarse grid spanning the entire molecular system and defining the global electrostatic environment and boundary conditions, and the small focus, which corresponds to a finer grid with higher spatial resolution, as needed to accurately capture electrostatics near charges and interfaces. For each focus level, APBS outputs files such as `dielx_L.dx`, `diely_L.dx`, `dielz_L.dx`, `kappa_L.dx`, `charge_L.dx` (and their analogous "_S" counterparts), which encode the protein-solvent dielectric boundary and ion-accessibility. These initial maps, prepared without a membrane, can then be modified by `draw_membrane2` before being used in a subsequent full APBS run, where the Poisson-Boltzmann equation is solved with the membrane explicitly represented.

An example command sequence, launched through manual execution on a terminal would look like this:

```
#Copy prepared PQR file to the name expected by the .in templates
cp input_structure.pqr TM.pqr

#Run dummy APBS to generate coefficient maps
apbs apbs_dummy.in > structure_dummy.out
```

Step 3 - Insert the membrane with draw_membrane2

After the dummy APBS run has produced the coefficient maps, the membrane is incorporated by editing the generated DX files using `draw_membrane2`, a utility provided with `APBSmem/BornProfiler`. For example, commands similar to those used in the pipeline are:

```
draw_membrane2 dielx_L.dx -18 36 10.0 0.0 0.15 23.2 17.5 > dielx_L.dx_draw.log
draw_membrane2 dielx_S.dx -18 36 10.0 0.0 0.15 23.2 17.5 > dielx_S.dx_draw.log
```

In these commands, the arguments define the geometric and physical properties of the inserted membrane. The first value specifies the z-coordinate (in Å) of the lower leaflet of the bilayer, effectively setting the vertical position of the membrane in the simulation box. The second value indicates the thickness of the bilayer, defining the width of the low-dielectric slab that represents the hydrophobic core of the membrane. The third argument sets the dielectric constant of the protein within the membrane region. The fourth value corresponds to the transmembrane potential, which is set to zero in this example. The fifth argument defines the bulk salt concentration in molar units, representing the ionic environment. Finally, the last two values indicate the radii of cone-shaped cutouts (upper and lower portion of the pore) in the membrane that impede the intrusion of the dielectric slab into the pore lumen, keeping the channel's interior solvent accessible. These parameters collectively ensure that the resulting coefficient maps accurately reflect both the presence of the membrane and the accessible solvent regions inside the protein pore.

Stage 4 - Final APBS run that reads the membrane-modified maps

After draw_membrane2 has edited the coefficient DX maps, a final full APBS run reads the modified maps and computes the electrostatic potential including the membrane contribution:

```
#Full membrane-included APBS run
apbs apbs_solv.in > structure_solv.out
```

The resulting pot_Sm.dx finer grid potential map can be loaded and visualized in different 3D protein viewer software, for instance, Pymol, allowing the electrostatic potential to be mapped onto the surface of the studied pore for detailed analysis.

While the initial setup of the parameters and conditions that regulate the APBS run can be quite laborious and require several attempts to optimize for each structure of interest, once the setup is completed, the pipeline allows multiple mutants of the same nanopore to be processed quickly. The workflow supports multiple PDB inputs and ionic concentrations, providing flexibility for diverse electrostatic conditions and producing outputs organized according to a user-defined directory structure. Because each APBS run requires a significant amount of RAM, for instance, the APBS simulations for Fig. 4 of the manuscript required $\simeq$ 24 GB, longer and finer grid runs, especially when applied to multiple models, are best executed on workstations or high-performance computing environments to ensure efficient and reliable calculations.

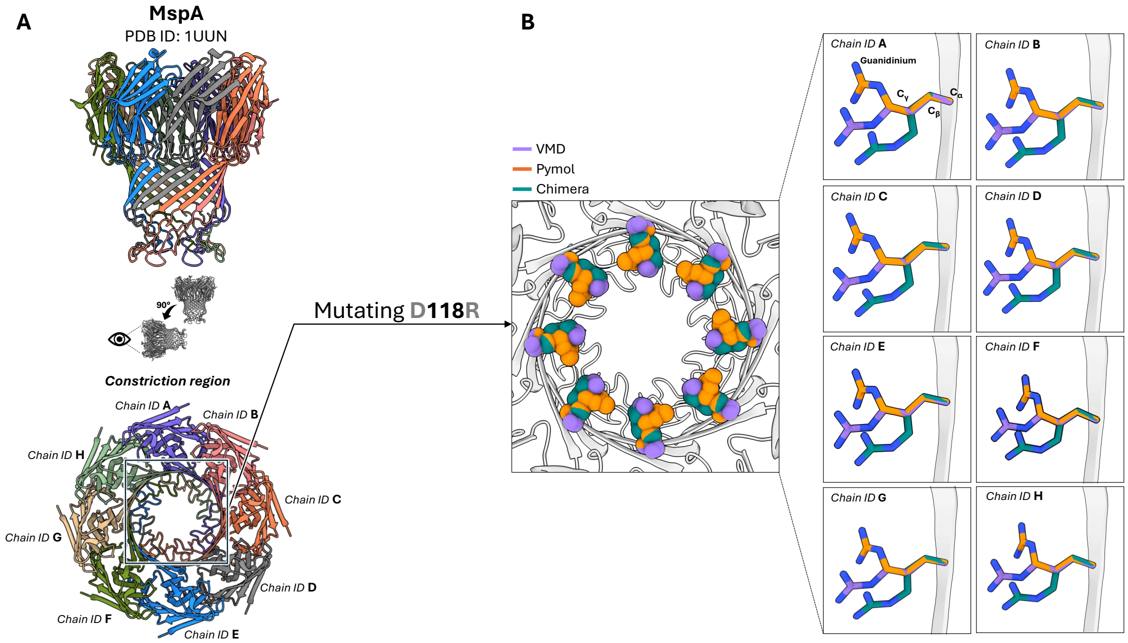

FIG. S1. **Differences in modeled rotamers of nanopore point mutations.** **(A)** Structural context of the mutation site within the Mycobacterium smegmatis porin A (MspA) nanopore. Top and side views of the MspA octamer (PDB ID: 1UUN [S17]) highlight the β-barrel region where the mutation was introduced. Each monomer is shown in a different color, and chain identifiers are labeled. The boxed region outlines the constriction of the pore. The mutation D118R is close to the constriction. **(B)** Comparison of the resulting arginine side chains modeled by VMD (purple), ChimeraX (teal), and PyMOL (orange). While the C_α and C_β atoms remain consistently positioned across all three models, substantial divergence emerges from the C_γ atom onward.

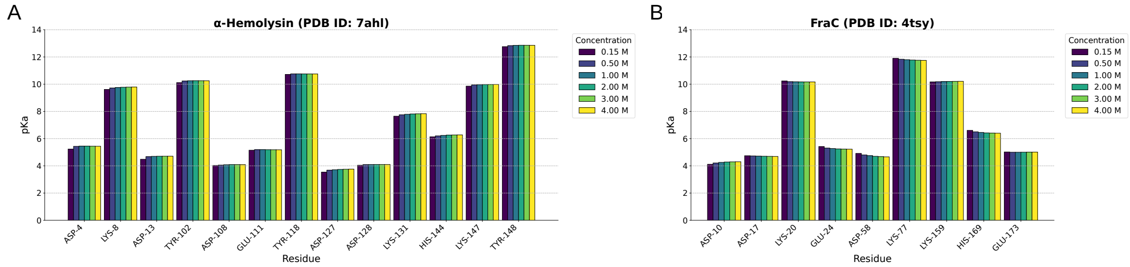

FIG. S2. **Effect of ionic concentration on pK_a predictions.** pK_a values calculated at different sality concentrations (0.15–4.0 M) for selected residues facing the pore lumen in α-hemolysin **(A)** and Fragaceatoxin C (FraC) **(B)**, same structures used in Fig. 2 of the manuscript. Each bar represents the pK_a at a specific salt concentration (color scale from dark to light indicates increasing ionic strength, the 0.15 M case is the one already reported in Fig. 2 of the manuscript). Calculations were performed using the H++ web server [S11]. The maximal difference between the minimal and maximal pK_a that we observed is 0.3 pK_a.

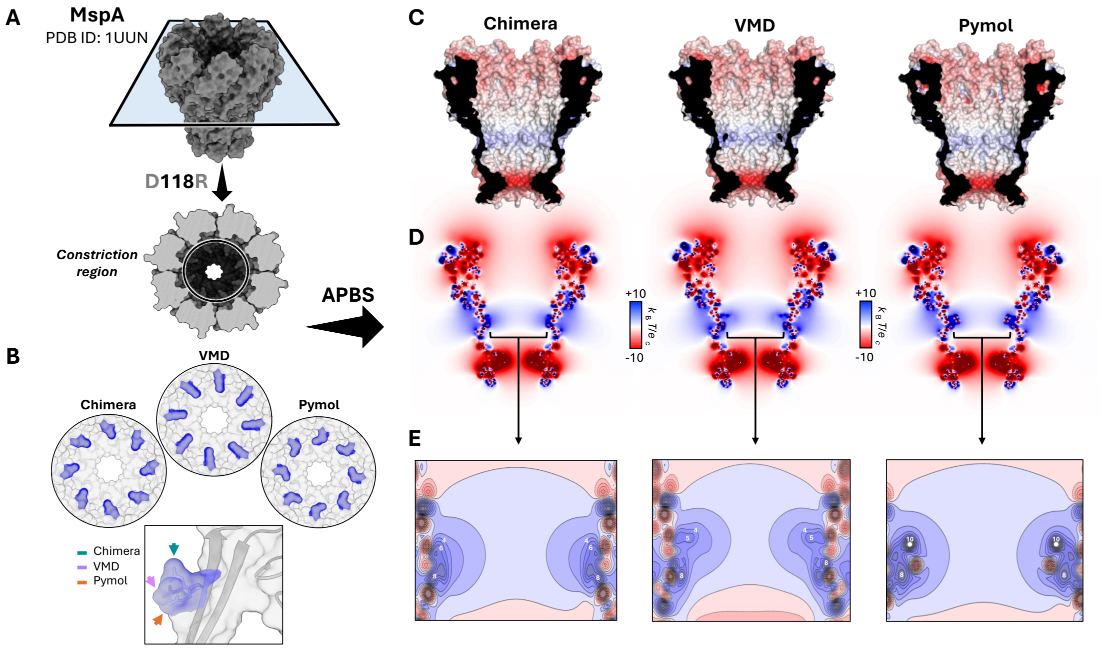

FIG. S3. **Comparative effects of D118R modeling on the electrostatic potential of MspA. (A)** Surface view of the MspA nanopore (PDB_ID: 1UUN) with a horizontal slice (light blue) cutting through the channel to reveal the constriction zone. **(B)** Axial perspective of the constriction, marking the D118R mutation site. The three panels display the introduced arginine side chains (blue) as modeled in Chimera, VMD, and PyMOL, highlighting program-specific differences in orientation and rendering. A zoomed view of a single mutated chain illustrates how conformational variability alters how the side chain protrudes into the pore cavity. **(C)** Electrostatic surface potential representations of the D118R variants, calculated with APBS and mapped onto the models produced by each software package. Regions of positive and negative potential are shown in blue and red, respectively. **(D)** Two-dimensional cross-sectional electrostatic maps corresponding to the models in panel C. These highlight that software-dependent conformations of Arg118 produce noticeable local changes in the electrostatic profile along the pore constriction. **(E)** Kernel density estimate (KDE) plots of the constriction region, drawn with isolines separated by 1 k_BT/e. This representation enables finer discrimination of electrostatic differences introduced by the distinct modeling approaches, revealing subtle variations that are less evident in the broader 2D potential slices.

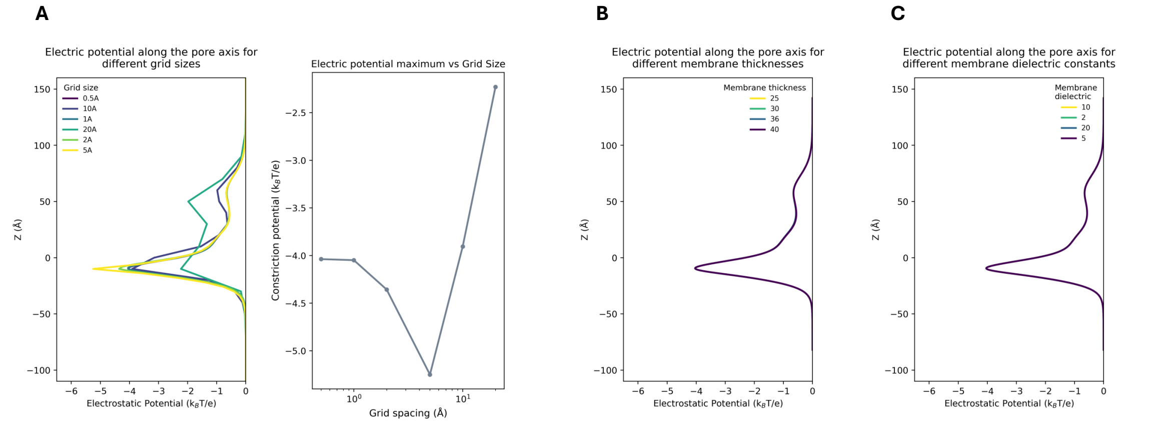

FIG. S4. **Sensitivity of the electrostatic potential along the nanopore axis to APBS parameters.** **(A)** Electrostatic potential evaluated along the nanopore axis using progressively finer APBS grid spacings, together with the convergence of the electrostatic potential at the characteristic constriction region as a function of grid spacing. Line plots report the axial electrostatic potential profiles, while the accompanying convergence analysis quantifies the potential value at the constriction as grid resolution is refined. Coarser grids produce deviations in peak magnitude and local features; however, the electrostatic potential converges rapidly with grid refinement, with negligible differences observed for grid spacings finer than 2 Å. **(B)** Effect of membrane-slab thickness on the electrostatic potential along the nanopore axis. Moderate variations in slab thickness do not produce appreciable changes in the electrostatic potential profile. Both the magnitude and spatial distribution of the potential along the pore axis remain stable, indicating robustness of the calculation with respect to reasonable membrane thickness choices. **(C)** Effect of membrane dielectric constant on the electrostatic potential along the nanopore axis. Variations in membrane dielectric preserve the overall shape and spatial features of the electrostatic potential profile. In panel **(B)** and **(C)**, on the scale of the graph, the curves overlap. Their difference is below $10^{-2}\,k_BT/e$.

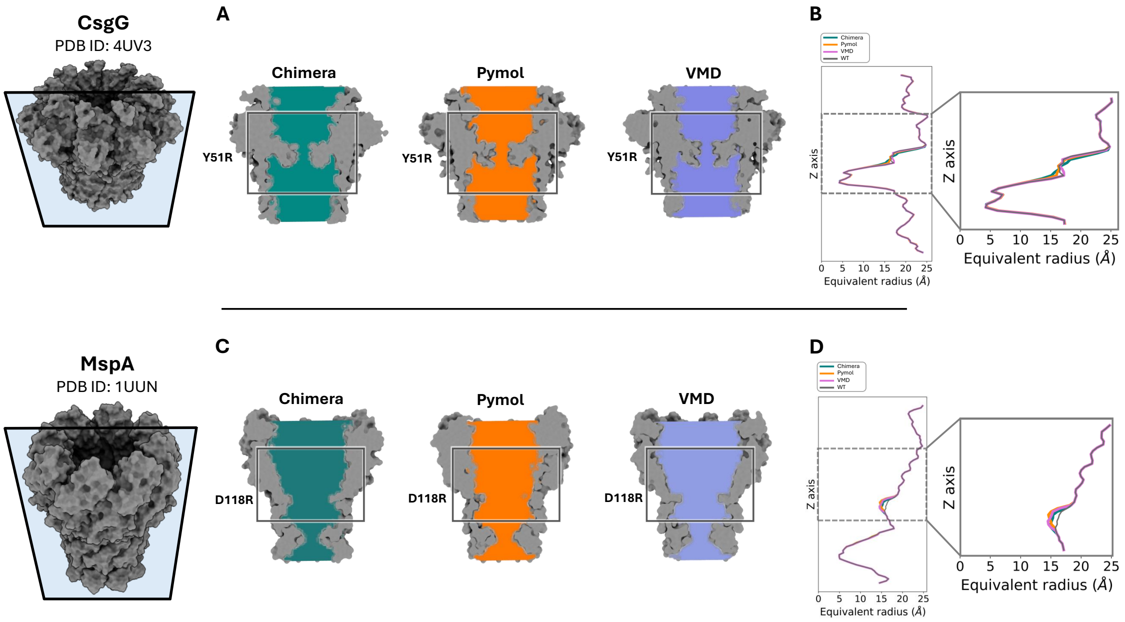

FIG. S5. **Estimating the volume of the nanopore lumen.** The internal volume of the nanopores was calculated using POVME 3.0 [S18] installed locally on a Unix-based system. Volume calculations were performed for both MspA (PDB ID: 1UUN) and CsgG (PDB ID: 4UV3), the latter modeled with AlphaFold3 as described in Supplementary Note 1. To define an inclusion shape that encompassed the entire internal volume of the nanopores, mass-weighted centroids were defined at the edges of both the extracellular and intracellular ends of each pore model. For MspA, the centroids were computed by first identifying two diametrically opposed monomers in the structure and selecting one representative residue near the edge of the pore in each monomer, both at the extracellular and intracellular sides. A mass-weighted centroid was then calculated between the pair of residues on each side, yielding two reference points aligned with the pore's central axis. For CsgG, residues at the extracellular and intracellular termini from all nine chains were selected to form two rings of residues, one at the top and one at the bottom of the nanopore. A mass-weighted centroid was then computed for each ring, providing two reference points that accurately represent the central pore axis across the full nonameric assembly. These centroid coordinates were then used to position two cylinders via the InclusionCylinder function in POVME: one cylinder extending from the extracellular side, and the other from the intracellular side, both converging toward the central constriction. This dual-cylinder setup ensured comprehensive sampling of the channel interior while adapting to variations in pore width along the z-axis. To further refine the inclusion region and prevent the incorporation of irrelevant volume outside the pore lumen, the convexHullExclusion option was enabled. Additionally, small ExclusionSpheres were manually placed in regions where the inclusion cylinders approached surface indentations or crevices. While no distinct side channels were present in these systems, this refinement step helped ensure that only the central lumen was measured and that small peripheral cavities not part of the true pore interior were excluded from the volume calculation. Default parameters were used for grid spacing and distance cutoffs in POVME. Specifically, with the default grid spacing of 1.0Å, the generated volumetric maps in .dx format consist of a 3D array of cubic voxels, each measuring 1Å× 1Å× 1Å. Each voxel stores a binary value, 1 when included within the defined pore volume, or 0 if excluded. These volumetric maps were visualized using VMD 1.9.4 by loading both the associated .pdb structure and the corresponding .dx grid. The internal volumes were then rendered as surfaces using ChimeraX [S4] and following a color scheme consistent with the one shown in Figure 2 of the manuscript. To quantitatively analyze the pore shape, a custom Python script was developed to extract the cross-sectional area and equivalent radius of the pore along the z-axis. The script reads the .dx file and processes each horizontal slice of the grid independently. For each z-slice, it identifies all occupied voxels and calculates the total cross-sectional area based on the known voxel size.

The equivalent radius for each slice is then computed by assuming a circular cross-section and determining the radius that would correspond to the measured area.

In this figure, panels A and C show z-axis slices of the mutated structures, with their internal volume rendered using the same coloring scheme as in Fig 1 of the manuscript and Fig S1. At this broader structural level, only faint differences are visible near the constriction region. By contrast, the equivalent-radius profiles shown in panels B and D more effectively capture the subtle yet measurable influence of local spatial rearrangements on pore geometry. In CsgG, the constriction region itself shows no appreciable difference between the wild-type and mutated structures; however, a contiguous segment adjacent to the constriction exhibits a slight variation in equivalent radius. In MspA, the observed change is more pronounced and aligns precisely with the location of the introduced mutations. Overall, the minimal variation detected suggests that, even when side-chain orientations differ, their impact on the lumen remains limited if they occupy similar spatial regions, thereby preserving the local pore geometry.

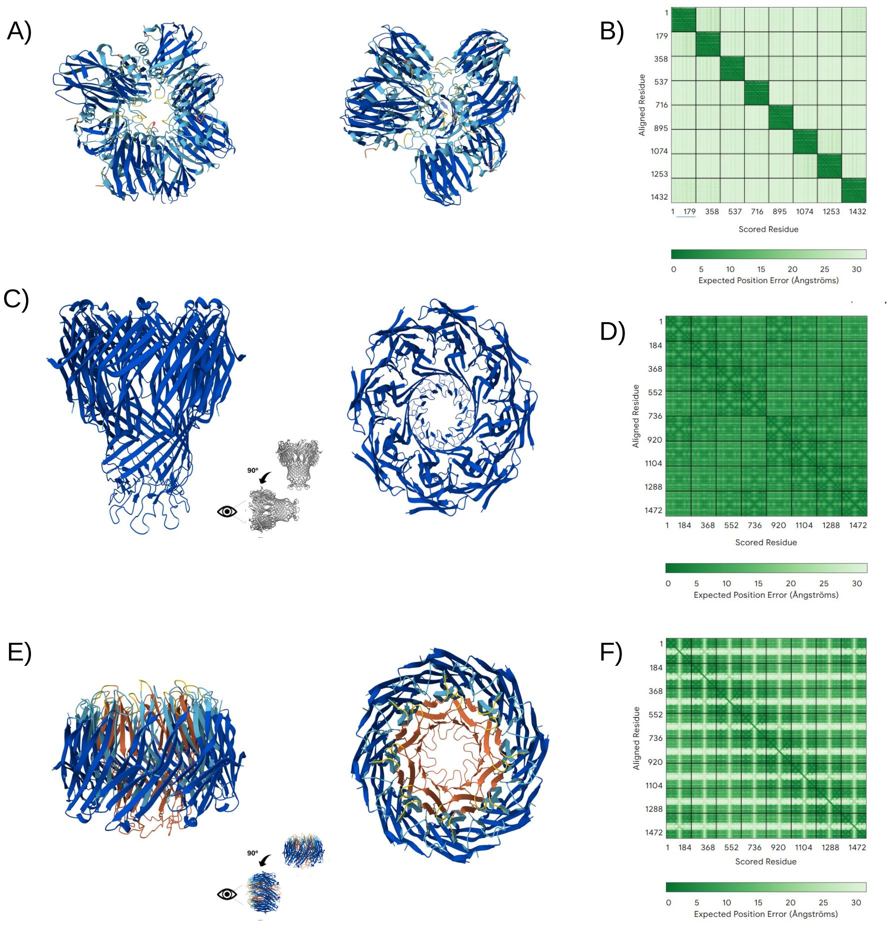

FIG. S6. **Additional data on MspA and FraC modelling with AlphaFold3.** **(A)** Side view and top view of predicted structure of FraC by AlphaFold3 colored according to the pLDDT (per-atom confidence) score of AlphaFold3: blue represents very high confidence (pLDDT $>$ 90), sky blue represents confident (70 $<$ pLDDT $<$ 90), yellow represents low confidence (50 $<$ pLDDT $<$ 70), red represents very low confidence (pLDDT $<$ 50). We used the sequence of PDB: 4TSY [S13] with Custom Template: Auto selection up to 03/02/2025 on AlphaFold3 Server version. The panel reports the same structure of panels C and D of Fig. 7 of the manuscript. PAE is reported in **(B)**. **(C)** Side view and top view of predicted structure of MspA colored according to the pLDDT (per-atom confidence) score of AlphaFold3. We used the sequence of MspA PDB:1UUN with a mutation L88R. PAE is reported in **(D)**. **(E)** Side view and top view of predicted structure of MspA colored according to the pLDDT (per-atom confidence) score of AlphaFold3. We used the sequence of MspA PDB:1UUN. PAE is reported in **(F)**. It is worth noting how a single residue can alter the structural prediction. In the case of the sequence present in the PDB entry 1UUN [S17] of MspA (E), both the PAE (F) and the predicted structure clearly indicate an incorrect fold, whereas the MspA variant mutated at residue 88 (C) yields a perfectly predicted structure (D). This highlights the need to use such tools with caution: even when templates and crystallographic structures are available, the prediction may still be wrong. In cases such as MspA, which is a very stable protein where a single mutation does not affect the overall nanopore structure, approaches like homology modeling may be more appropriate than deep learning-based predictions.

SUPPLEMENTARY TABLE S1: OVERVIEW ON ROTAMER SELECTION STRATEGIES IN MUTATION MODELING TOOLS

Summary table of the rotamer sources and selection strategies employed by the three software packages examined for introducing mutations in nanopores. The table also provides a brief overview of the principal strengths and limitations of each tool, offering general guidance on their suitability for different modeling scenarios.

Tool	Side chain source	Side chain rotamer selection	Strengths	Limitations
**VMD**	Psfgen automatic structure building tool	Automatic	GUI or Tcl scripting	No clash detection or environmental awareness
**PyMOL**	Dunbrack backbone-dependent/independent library	Automatic; Manual	Interactive visualization	No quantitative clash or energy scoring
**ChimeraX**	Dunbrack backbone-dependent/independent library	Automatic; Manual	Quantitative clash scoring; Interactive visualization	Static geometry; no energy minimization

SUPPLEMENTARY TABLE S2: PROPKA3-PREDICTED PKA VALUES FOR α-HEMOLYSIN.

TABLE S2: PROPKA3-predicted pKa values for α-hemolysin.

ID	Name	Mean±SD	A	B	C	D	E	F	G
1	N+	7.87 ± 0.17	7.88	7.65	7.95	8.11	7.72	8.02	7.73
2	ASP	3.95 ± 0.62	4.76	3.70	3.14	3.34	3.90	4.17	4.64
4	ASP	3.25 ± 0.15	3.07	3.19	3.20	3.25	3.37	3.18	3.52
8	LYS	10.18 ± 0.19	10.40	10.07	10.22	9.84	10.32	10.13	10.31
13	ASP	5.61 ± 0.15	5.65	5.33	5.54	5.76	5.54	5.71	5.74
21	LYS	10.23 ± 0.13	10.41	10.16	10.22	10.11	10.09	10.25	10.38
24	ASP	1.11 ± 0.30	1.25	1.39	1.07	0.46	1.25	1.12	1.22
28	TYR	11.20 ± 0.06	11.16	11.21	11.22	11.13	11.24	11.30	11.16
29	ASP	2.14 ± 0.16	2.28	2.10	2.36	2.00	2.24	2.10	1.92
30	LYS	10.54 ± 0.17	10.48	10.49	10.82	–	10.53	10.36	–
31	GLU	4.77 ± 0.05	4.70	4.75	4.77	4.72	4.85	4.80	4.77
35	HIS	4.18 ± 0.05	4.11	4.18	4.13	4.22	4.22	4.26	4.17
36	LYS	9.62 ± 0.31	9.26	10.03	9.52	9.89	9.20	9.78	9.65
37	LYS	11.25 ± 0.15	11.40	11.35	11.34	11.16	10.96	11.26	11.26
40	TYR	12.17 ± 0.14	12.10	12.28	12.18	12.31	12.06	12.32	11.97
44	ASP	2.56 ± 0.48	3.12	2.94	2.01	3.08	2.35	2.40	2.05
45	ASP	3.71 ± 0.18	3.89	3.50	3.70	3.92	3.51	3.84	3.62
46	LYS	10.89 ± 0.25	10.59	10.79	11.14	10.60	11.11	11.17	10.85
48	HIS	5.99 ± 0.14	6.11	5.84	5.83	6.17	5.88	6.11	5.97
50	LYS	9.59 ± 0.54	9.73	9.23	9.05	9.23	9.68	10.68	9.50
51	LYS	10.07 ± 0.58	9.69	9.79	10.12	9.81	10.21	9.58	11.29
56	ARG	12.28 ± 0.30	12.21	11.97	12.62	12.26	12.62	11.85	12.40
58	LYS	8.96 ± 0.17	9.09	8.72	9.04	9.11	8.73	9.03	9.01
65	TYR	14.11 ± 0.28	13.68	14.08	14.39	14.38	14.37	13.98	13.86
66	ARG	12.07 ± 0.18	–	11.82	12.14	12.17	12.00	11.97	12.32
68	TYR	12.39 ± 0.51	11.43	12.68	12.34	13.05	12.20	12.34	12.72
70	GLU	4.26 ± 0.52	4.57	4.34	3.70	4.56	3.90	5.06	3.67
71	GLU	4.38 ± 0.18	4.57	4.47	4.10	4.16	4.39	4.47	4.48
75	LYS	10.31 ± 0.42	–	9.48	10.32	10.52	10.54	10.52	10.50
85	LYS	10.67 ± 0.18	10.64	10.54	10.71	10.56	11.07	10.56	10.63
92	ASP	3.81 ± 0.17	3.92	3.70	4.00	3.77	3.49	3.89	3.87
94	GLU	4.96 ± 0.11	4.94	5.05	4.78	5.07	4.99	5.05	4.83

ID	Name	Mean±SD	A	B	C	D	E	F	G
100	ASP	0.43 ± 0.30	0.06	0.26	1.00	0.26	0.48	0.41	0.54
101	TYR	14.51 ± 0.65	13.97	15.15	15.06	13.99	13.56	14.91	14.91
102	TYR	10.76 ± 0.01	10.76	10.74	10.75	10.77	10.78	10.76	10.74
104	ARG	14.27 ± 0.07	14.22	14.36	14.29	14.20	14.32	14.32	14.18
108	ASP	4.91 ± 0.25	4.78	4.87	4.88	4.80	5.46	4.87	4.74
110	LYS	9.34 ± 0.17	9.10	9.55	9.42	9.48	9.44	9.22	9.17
111	GLU	3.87 ± 0.75	3.91	4.27	3.63	2.70	4.81	3.22	4.54
112	TYR	12.02 ± 0.31	11.49	12.40	11.85	12.20	12.02	11.90	12.26
118	TYR	11.50 ± 0.24	11.28	11.69	11.40	11.63	11.72	11.10	11.65
127	ASP	3.70 ± 0.41	3.55	3.13	3.82	4.21	3.57	4.24	3.40
128	ASP	4.18 ± 0.20	4.37	4.08	4.07	3.98	4.54	4.18	4.04
131	LYS	10.43 ± 0.43	11.09	10.08	10.73	10.11	10.49	9.88	10.61
144	HIS	4.53 ± 0.19	4.55	4.57	4.46	4.54	4.22	4.51	4.86
147	LYS	11.20 ± 0.33	11.02	11.35	11.10	11.62	11.21	11.49	10.62
148	TYR	14.03 ± 0.25	14.15	14.14	14.17	13.84	13.72	13.79	14.41
152	ASP	5.92 ± 0.58	6.00	6.06	6.27	5.89	6.01	4.70	6.52
154	LYS	9.09 ± 0.14	9.21	8.84	9.00	9.26	9.04	9.10	9.16
158	GLU	4.75 ± 0.41	5.11	4.44	4.26	4.76	4.31	5.22	5.13
162	ASP	3.39 ± 0.15	3.53	3.44	3.49	3.12	3.27	3.48	3.42
163	LYS	9.74 ± 0.23	9.33	9.82	9.81	9.78	9.97	9.53	9.94
164	LYS	9.59 ± 0.65	9.10	8.95	10.19	10.19	9.72	10.26	8.74
168	LYS	9.82 ± 0.33	9.58	10.10	10.28	10.06	9.54	9.43	9.72
182	TYR	11.49 ± 0.31	11.37	12.03	11.56	11.24	11.26	11.22	11.76
183	ASP	4.23 ± 0.48	3.93	3.63	4.00	4.92	4.51	3.92	4.72
184	ARG	13.88 ± 0.22	14.11	13.84	14.09	13.57	14.00	13.60	13.94
185	ASP	2.08 ± 0.38	1.73	2.38	2.65	1.95	1.59	2.29	1.96
191	TYR	9.46 ± 1.00	9.94	10.61	10.04	8.22	8.87	8.22	10.30
198	LYS	9.79 ± 0.13	9.66	9.90	10.02	9.73	9.70	9.70	9.85
200	ARG	12.51 ± 0.41	12.28	12.33	11.92	12.99	12.30	12.96	12.79
205	LYS	11.03 ± 0.63	9.91	10.65	11.60	11.13	11.52	10.76	11.64
208	ASP	3.73 ± 0.18	3.83	3.83	3.91	3.38	3.70	3.67	3.80
212	ASP	3.90 ± 0.22	3.83	3.66	3.96	3.74	4.15	3.71	4.23
215	LYS	11.38 ± 0.35	11.42	11.89	11.44	11.09	10.82	11.66	11.36
227	ASP	3.53 ± 0.12	3.34	3.71	3.42	3.60	3.54	3.48	3.59
235	ASP	3.45 ± 0.26	3.71	3.11	3.61	3.77	3.39	3.15	3.38

ID	Name	Mean±SD	A	B	C	D	E	F	G
236	ARG	12.59 ± 0.09	12.52	12.58	12.64	12.67	12.69	12.61	12.42
237	LYS	10.47 ± 0.03	10.47	10.50	10.47	10.48	10.48	10.42	10.50
240	LYS	10.55 ± 0.32	10.32	10.43	10.41	–	10.50	11.19	10.48
246	ASP	3.19 ± 0.38	3.78	3.70	2.90	3.05	3.07	2.99	2.84
249	TYR	14.07 ± 0.43	13.63	13.74	14.31	14.46	13.52	14.54	14.31
250	GLU	4.84 ± 0.46	4.93	4.23	5.27	4.29	5.26	4.63	5.28
251	ARG	11.04 ± 0.19	11.07	11.17	11.08	11.08	11.10	10.61	11.17
253	ARG	12.68 ± 0.29	12.51	13.11	12.32	12.47	12.93	12.55	12.85
254	ASP	4.44 ± 0.14	4.35	4.37	4.48	4.38	4.43	4.73	4.32
255	ASP	3.69 ± 0.59	3.16	3.47	3.00	3.66	3.69	4.19	4.69
256	TYR	14.01 ± 0.19	14.09	13.87	13.91	13.79	13.88	14.32	14.18
259	HIS	4.72 ± 0.28	5.23	4.29	4.70	4.66	4.66	4.68	4.79
266	LYS	9.07 ± 0.50	8.05	9.30	8.85	9.11	9.24	9.52	9.45
271	LYS	10.99 ± 0.27	10.81	11.01	11.36	11.33	10.94	10.85	10.62
272	ASP	3.28 ± 0.22	3.00	3.40	3.16	3.67	3.30	3.31	3.10
273	LYS	11.21 ± 0.36	11.64	11.36	11.25	11.41	11.28	10.99	10.51
276	ASP	2.85 ± 0.35	2.95	2.31	3.18	2.97	2.53	3.30	2.71
277	ARG	12.80 ± 0.38	12.65	13.12	12.48	13.40	12.47	13.03	12.46
280	GLU	4.47 ± 0.66	5.12	3.23	4.65	3.91	4.88	4.71	4.81
281	ARG	13.79 ± 0.40	13.22	13.21	14.05	13.95	13.98	13.97	14.17
282	TYR	13.87 ± 0.62	14.39	14.77	13.62	13.50	14.32	13.32	13.17
283	LYS	10.30 ± 0.15	10.33	10.09	10.26	10.32	10.54	–	10.26
285	ASP	2.95 ± 0.40	3.10	3.02	3.33	3.11	3.13	2.86	2.10
287	GLU	4.69 ± 0.35	4.73	4.83	4.84	4.78	4.84	3.91	4.89
288	LYS	11.26 ± 0.45	10.82	10.72	10.87	11.40	11.58	11.55	11.89
289	GLU	5.71 ± 0.60	6.10	6.04	5.92	5.88	5.51	6.06	4.43
290	GLU	4.37 ± 0.45	4.91	4.67	4.84	4.04	3.78	3.96	4.41
293	C-	3.42 ± 0.04	3.44	3.50	3.43	3.37	3.39	3.43	3.41

SUPPLEMENTARY TABLE S3: H++-PREDICTED PKA VALUES FOR α-HEMOLYSIN.

TABLE S3: H++ predicted pKa values for α-hemolysin.

ID	Name	Mean±SD	A	B	C	D	E	F	G
1	N+	6.72 ± 0.18	6.55	6.55	6.68	6.61	6.73	7.00	6.95
2	ASP	4.71 ± 0.35	4.82	4.65	4.79	4.24	4.30	4.92	5.24
4	ASP	5.33 ± 0.26	5.15	5.19	5.18	5.10	5.58	5.78	5.31
8	LYS	9.65 ± 0.24	9.76	9.43	9.46	10.07	9.80	9.43	9.55
13	ASP	4.61 ± 0.15	4.42	4.66	4.55	4.60	4.49	4.70	4.86
21	LYS	10.80 ± 0.10	10.75	10.77	10.92	10.84	10.92	10.78	10.63
24	ASP	7.38 ± 0.40	6.57	7.84	7.48	7.53	7.42	7.28	7.54
28	TYR	11.04 ± 0.24	11.07	11.03	10.57	11.09	11.04	11.35	11.16
29	ASP	2.91 ± 0.22	2.95	2.86	3.07	2.87	2.55	3.25	2.79
30	LYS	10.16 ± 0.12	10.14	10.24	9.94	10.23	10.05	10.24	10.26
31	GLU	4.39 ± 0.05	4.46	4.40	4.36	4.32	4.41	4.45	4.37
35	HIS	4.88 ± 0.71	4.86	5.61	5.49	5.39	4.91	3.71	4.17
36	LYS	9.27 ± 0.23	9.41	9.35	9.10	8.83	9.47	9.41	9.35
37	LYS	9.59 ± 0.50	8.61	9.72	10.01	9.46	10.13	9.45	9.74
40	TYR	11.86 ± 0.47	11.32	12.59	11.53	12.24	11.54	12.17	11.65
44	ASP	6.01 ± 0.08	5.97	5.99	5.99	6.01	5.90	6.04	6.17
45	ASP	4.76 ± 0.19	4.98	4.69	4.67	4.74	4.62	4.55	5.05
46	LYS	10.44 ± 0.13	10.55	10.59	10.20	10.41	10.54	10.38	10.42
48	HIS	5.39 ± 0.33	5.90	5.52	4.89	5.53	5.07	5.49	5.32
50	LYS	9.75 ± 0.50	9.46	9.99	9.79	10.02	10.16	10.08	8.74
51	LYS	9.98 ± 0.29	10.13	10.17	9.51	9.98	10.15	10.27	9.67
56	ARG	10.93 ± 0.30	11.28	10.85	11.00	11.20	10.63	10.46	11.08
58	LYS	11.22 ± 0.47	10.25	11.38	11.34	11.19	11.31	11.33	11.78
65	TYR	14.35 ± 0.25	14.14	14.43	14.02	14.77	14.16	14.44	14.46
66	ARG	11.95 ± 0.13	12.07	11.69	11.98	11.96	11.89	11.98	12.05
68	TYR	11.58 ± 0.66	12.12	11.01	11.79	11.82	10.36	12.03	11.97
70	GLU	4.82 ± 0.37	4.60	4.44	4.79	4.60	4.78	5.58	4.93
71	GLU	4.81 ± 0.91	4.25	4.15	4.40	5.26	4.29	6.70	4.63
75	LYS	9.75 ± 0.40	9.10	9.25	9.98	9.98	9.94	10.01	10.02
85	LYS	9.63 ± 0.18	9.62	9.63	9.89	9.84	9.41	9.50	9.49
92	ASP	4.19 ± 0.24	4.21	4.42	4.51	3.76	4.20	4.20	4.05
94	GLU	5.24 ± 0.40	4.73	5.08	5.46	5.38	5.96	5.01	5.09

ID	Name	Mean±SD	A	B	C	D	E	F	G
100	ASP	9.14 ± 0.40	9.45	9.51	9.04	9.11	9.04	9.46	8.36
101	TYR	14.34 ± 0.90	15.79	14.63	14.66	12.89	13.81	13.97	14.63
102	TYR	10.16 ± 0.09	10.10	10.12	10.11	10.05	10.16	10.31	10.25
104	ARG	12.67 ± 0.23	12.64	12.40	12.58	12.58	12.80	13.11	12.55
108	ASP	4.06 ± 0.16	4.22	4.03	3.91	4.09	3.81	4.09	4.26
110	LYS	9.51 ± 0.78	9.72	10.41	9.00	10.48	9.65	8.45	8.87
111	GLU	5.16 ± 0.19	5.17	5.03	5.02	5.29	4.88	5.32	5.42
112	TYR	12.09 ± 0.60	11.20	11.75	12.80	12.21	12.23	11.63	12.79
118	TYR	10.82 ± 0.14	11.00	10.77	10.98	10.66	10.63	10.85	10.83
127	ASP	3.38 ± 0.33	3.36	3.30	3.22	3.94	3.61	3.31	2.90
128	ASP	4.01 ± 0.18	4.16	4.16	3.82	4.25	3.87	3.81	4.02
131	LYS	7.60 ± 0.77	7.96	7.89	6.96	6.26	7.64	8.63	7.89
144	HID	6.57 ± 0.35	6.60	6.78	6.49	6.88	6.39	5.92	6.94
147	LYS	9.83 ± 0.21	9.93	9.99	9.56	9.97	9.50	9.89	9.97
148	TYR	12.78 ± 1.39	14.00	12.66	10.86	13.62	13.46	10.85	13.99
152	ASP	5.05 ± 0.30	5.04	5.31	5.32	4.55	5.26	5.11	4.75
154	LYS	9.93 ± 0.18	10.03	10.04	9.57	9.86	9.90	10.03	10.06
158	GLU	5.91 ± 0.27	5.97	5.32	5.96	6.15	5.95	5.99	5.99
162	ASP	4.52 ± 0.49	5.10	4.66	4.33	3.67	4.55	4.29	5.02
163	LYS	10.20 ± 0.26	10.38	10.27	10.44	9.77	9.93	10.43	10.16
164	LYS	10.05 ± 0.19	9.91	9.96	10.17	10.04	10.04	10.42	9.84
168	LYS	9.72 ± 0.15	9.78	9.60	9.50	9.70	9.94	9.83	9.65
182	TYR	11.49 ± 0.55	11.36	11.58	12.12	11.98	10.71	11.83	10.85
183	ASP	3.51 ± 0.36	4.07	3.15	3.27	3.10	3.83	3.54	3.61
184	ARG	9.99 ± 0.15	10.25	9.93	9.85	9.89	10.13	10.03	9.85
185	ASP	4.63 ± 0.20	4.74	4.96	4.49	4.46	4.80	4.45	4.51
191	TYR	10.70 ± 0.24	11.07	10.51	10.73	10.90	10.78	10.37	10.54
198	LYS	10.26 ± 0.19	10.45	10.43	9.86	10.29	10.21	10.30	10.25
200	ARG	11.97 ± 0.40	11.55	12.09	12.67	12.03	11.57	12.18	11.73
205	LYS	9.54 ± 0.99	7.38	10.28	10.04	9.49	9.94	10.05	9.57
208	ASP	4.32 ± 0.14	4.14	4.36	4.29	4.54	4.21	4.43	4.25
212	ASP	4.03 ± 0.20	3.95	4.16	4.22	3.97	3.89	4.29	3.72
215	LYS	8.90 ± 0.20	8.92	9.04	8.81	8.98	8.94	9.09	8.49
227	ASP	5.23 ± 0.21	5.63	5.01	5.17	5.25	5.04	5.34	5.15
235	ASP	4.28 ± 0.33	4.12	4.11	4.53	4.24	4.27	4.87	3.85

ID	Name	Mean±SD	A	B	C	D	E	F	G
236	ARG	12.77 ± 0.18	13.09	12.87	12.68	12.58	12.64	12.63	12.87
237	LYS	10.42 ± 0.04	10.37	10.43	10.46	10.46	10.41	10.45	10.36
240	LYS	9.81 ± 0.45	8.98	10.24	9.99	10.03	9.46	9.80	10.20
246	ASP	5.23 ± 0.23	5.55	5.08	4.97	5.05	5.13	5.31	5.49
249	TYR	15.44 ± 0.31	15.47	15.02	15.29	15.54	15.19	15.65	15.95
250	GLU	5.65 ± 0.31	6.12	5.70	5.77	5.77	5.14	5.43	5.62
251	ARG	11.93 ± 0.39	11.83	11.77	12.64	11.84	12.05	12.05	11.35
253	ARG	11.46 ± 0.24	11.22	11.57	11.79	11.39	11.47	11.68	11.12
254	ASP	2.74 ± 0.72	2.25	2.45	2.51	2.73	2.67	4.33	2.24
255	ASP	4.07 ± 0.36	4.36	4.03	3.51	4.37	3.76	4.52	3.98
256	TYR	14.02 ± 0.35	13.88	14.22	14.32	14.30	13.54	14.29	13.59
259	HIS	6.15 ± 0.20	6.13	6.13	6.23	6.37	6.33	6.12	5.76
266	LYS	10.39 ± 0.17	10.24	10.51	10.47	10.47	10.05	10.47	10.50
271	LYS	9.43 ± 0.14	9.41	9.45	9.46	9.45	9.70	9.30	9.24
272	ASP	4.01 ± 0.14	3.90	4.03	3.91	4.21	3.86	4.17	4.00
273	LYS	9.63 ± 0.23	9.32	9.68	9.64	9.80	9.32	9.91	9.71
276	ASP	5.62 ± 0.34	5.04	5.58	5.67	5.95	5.39	6.04	5.64
277	ARG	11.60 ± 0.24	11.72	11.97	11.42	11.68	11.50	11.21	11.70
280	GLU	5.48 ± 0.32	5.59	5.54	6.04	5.58	5.03	5.29	5.30
281	ARG	11.64 ± 0.14	11.62	11.42	11.67	11.68	11.84	11.54	11.72
282	TYR	13.24 ± 0.47	12.94	13.90	13.85	13.16	12.93	13.26	12.67
283	LYS	10.13 ± 0.24	10.12	10.45	9.75	10.22	10.33	9.92	10.12
285	ASP	3.71 ± 0.32	3.46	3.65	3.73	3.33	3.77	4.35	3.70
287	GLU	4.70 ± 0.44	4.41	4.77	4.36	4.48	4.57	5.66	4.69
288	LYS	10.10 ± 0.30	10.19	10.30	10.17	10.14	10.29	10.15	9.44
289	GLU	5.93 ± 0.18	5.92	6.04	5.69	5.72	6.22	5.96	5.96
290	GLU	4.93 ± 0.27	4.56	4.88	4.74	4.81	5.38	5.07	5.07

SUPPLEMENTARY TABLE S4: PROPKA3 AND H++ COMPARISON OF PKA VALUES FOR α-HEMOLYSIN.

TABLE S4: Comparison of pK_a values between PROPKA3 and H++

ID	Residue	PROPKA3 ± SD	H++ ± SD	|Δ|
2	ASP	3.95 ± 0.62	4.71 ± 0.35	0.76
4	ASP	3.25 ± 0.15	5.33 ± 0.26	2.07
8	LYS	10.18 ± 0.19	9.65 ± 0.24	0.54
13	ASP	5.61 ± 0.15	4.61 ± 0.15	1.00
21	LYS	10.23 ± 0.13	10.80 ± 0.10	0.57
24	ASP	1.11 ± 0.30	7.38 ± 0.40	6.27
28	TYR	11.20 ± 0.06	11.04 ± 0.24	0.16
29	ASP	2.14 ± 0.16	2.91 ± 0.22	0.76
30	LYS	10.54 ± 0.17	10.16 ± 0.12	0.38
31	GLU	4.77 ± 0.05	4.39 ± 0.05	0.37
35	HIS	4.18 ± 0.05	4.88 ± 0.71	0.69
36	LYS	9.62 ± 0.31	9.27 ± 0.23	0.34
37	LYS	11.25 ± 0.15	9.59 ± 0.50	1.66
40	TYR	12.17 ± 0.14	11.86 ± 0.47	0.31
44	ASP	2.56 ± 0.48	6.01 ± 0.08	3.45
45	ASP	3.71 ± 0.18	4.76 ± 0.19	1.05
46	LYS	10.89 ± 0.25	10.44 ± 0.13	0.45
48	HIS	5.99 ± 0.14	5.39 ± 0.33	0.60
50	LYS	9.59 ± 0.54	9.75 ± 0.50	0.16
51	LYS	10.07 ± 0.58	9.98 ± 0.29	0.09
56	ARG	12.28 ± 0.30	10.93 ± 0.30	1.35
58	LYS	8.96 ± 0.17	11.22 ± 0.47	2.26
65	TYR	14.11 ± 0.28	14.35 ± 0.25	0.24
66	ARG	12.07 ± 0.18	11.95 ± 0.13	0.12
68	TYR	12.39 ± 0.51	11.58 ± 0.66	0.81
70	GLU	4.26 ± 0.52	4.82 ± 0.37	0.56
71	GLU	4.38 ± 0.18	4.81 ± 0.91	0.44
75	LYS	10.31 ± 0.42	9.75 ± 0.40	0.56
85	LYS	10.67 ± 0.18	9.63 ± 0.18	1.05
92	ASP	3.81 ± 0.17	4.19 ± 0.24	0.39
94	GLU	4.96 ± 0.11	5.24 ± 0.40	0.28
100	ASP	0.43 ± 0.30	9.14 ± 0.40	8.71

ID	Residue	PROPKA3 ± SD	H++ ± SD	|Δ|
101	TYR	14.51 ± 0.65	14.34 ± 0.90	0.17
102	TYR	10.76 ± 0.01	10.16 ± 0.09	0.60
104	ARG	14.27 ± 0.07	12.67 ± 0.23	1.60
108	ASP	4.91 ± 0.25	4.06 ± 0.16	0.86
110	LYS	9.34 ± 0.17	9.51 ± 0.78	0.17
111	GLU	3.87 ± 0.75	5.16 ± 0.19	1.29
112	TYR	12.02 ± 0.31	12.09 ± 0.60	0.07
118	TYR	11.50 ± 0.24	10.82 ± 0.14	0.68
127	ASP	3.70 ± 0.41	3.38 ± 0.33	0.33
128	ASP	4.18 ± 0.20	4.01 ± 0.18	0.17
131	LYS	10.43 ± 0.43	7.60 ± 0.77	2.82
147	LYS	11.20 ± 0.33	9.83 ± 0.21	1.37
148	TYR	14.03 ± 0.25	12.78 ± 1.39	1.25
152	ASP	5.92 ± 0.58	5.05 ± 0.30	0.87
154	LYS	9.09 ± 0.14	9.93 ± 0.18	0.84
158	GLU	4.75 ± 0.41	5.91 ± 0.27	1.16
162	ASP	3.39 ± 0.15	4.52 ± 0.49	1.12
163	LYS	9.74 ± 0.23	10.20 ± 0.26	0.46
164	LYS	9.59 ± 0.65	10.05 ± 0.19	0.46
168	LYS	9.82 ± 0.33	9.72 ± 0.15	0.10
182	TYR	11.49 ± 0.31	11.49 ± 0.55	0.00
183	ASP	4.23 ± 0.48	3.51 ± 0.36	0.73
184	ARG	13.88 ± 0.22	9.99 ± 0.15	3.89
185	ASP	2.08 ± 0.38	4.63 ± 0.20	2.55
191	TYR	9.46 ± 1.00	10.70 ± 0.24	1.24
198	LYS	9.79 ± 0.13	10.26 ± 0.19	0.46
200	ARG	12.51 ± 0.41	11.97 ± 0.40	0.54
205	LYS	11.03 ± 0.63	9.54 ± 0.99	1.49
208	ASP	3.73 ± 0.18	4.32 ± 0.14	0.59
212	ASP	3.90 ± 0.22	4.03 ± 0.20	0.13
215	LYS	11.38 ± 0.35	8.90 ± 0.20	2.49
227	ASP	3.53 ± 0.12	5.23 ± 0.21	1.70
235	ASP	3.45 ± 0.26	4.28 ± 0.33	0.84
236	ARG	12.59 ± 0.09	12.77 ± 0.18	0.18
237	LYS	10.47 ± 0.03	10.42 ± 0.04	0.05

ID	Residue	PROPKA3 ± SD	H++ ± SD	$\|\Delta\|$
240	LYS	10.55 ± 0.32	9.81 ± 0.45	0.74
246	ASP	3.19 ± 0.38	5.23 ± 0.23	2.04
249	TYR	14.07 ± 0.43	15.44 ± 0.31	1.37
250	GLU	4.84 ± 0.46	5.65 ± 0.31	0.81
251	ARG	11.04 ± 0.19	11.93 ± 0.39	0.89
253	ARG	12.68 ± 0.29	11.46 ± 0.24	1.21
254	ASP	4.44 ± 0.14	2.74 ± 0.72	1.70
255	ASP	3.69 ± 0.59	4.07 ± 0.36	0.38
256	TYR	14.01 ± 0.19	14.02 ± 0.35	0.02
259	HIS	4.72 ± 0.28	6.15 ± 0.20	1.44
266	LYS	9.07 ± 0.50	10.39 ± 0.17	1.31
271	LYS	10.99 ± 0.27	9.43 ± 0.14	1.56
272	ASP	3.28 ± 0.22	4.01 ± 0.14	0.73
273	LYS	11.21 ± 0.36	9.63 ± 0.23	1.58
276	ASP	2.85 ± 0.35	5.62 ± 0.34	2.77
277	ARG	12.80 ± 0.38	11.60 ± 0.24	1.20
280	GLU	4.47 ± 0.66	5.48 ± 0.32	1.01
281	ARG	13.79 ± 0.40	11.64 ± 0.14	2.15
282	TYR	13.87 ± 0.62	13.24 ± 0.47	0.63
283	LYS	10.30 ± 0.15	10.13 ± 0.24	0.17
285	ASP	2.95 ± 0.40	3.71 ± 0.32	0.76
287	GLU	4.69 ± 0.35	4.70 ± 0.44	0.02
288	LYS	11.26 ± 0.45	10.10 ± 0.30	1.16
289	GLU	5.71 ± 0.60	5.93 ± 0.18	0.22
290	GLU	4.37 ± 0.45	4.93 ± 0.27	0.56

SUPPLEMENTARY TABLE S5: PROPKA3-PREDICTED PKA VALUES FOR FRAC.

TABLE S5: PROPKA3-predicted pKa values for FraC.

ID	Name	Mean±SD	A	B	C	D	E	F	G	H
4	N+	7.86 ± 0.01	7.86	7.85	7.85	7.86	7.86	7.85	7.85	7.86
10	ASP	4.25 ± 0.14	4.36	4.12	4.40	4.13	4.36	4.12	4.40	4.13
17	ASP	3.76 ± 0.50	2.95	4.01	4.09	3.98	2.95	4.01	4.09	3.98
20	LYS	10.98 ± 0.33	11.51	10.82	10.76	10.82	11.51	10.82	10.76	10.82
24	GLU	4.55 ± 0.07	4.66	4.51	4.50	4.54	4.66	4.51	4.50	4.54
30	LYS	10.25 ± 0.01	10.24	10.24	10.26	10.24	10.24	10.24	10.26	10.24
31	ARG	11.44 ± 0.01	11.44	11.42	11.45	11.44	11.44	11.42	11.45	11.44
32	LYS	9.74 ± 0.00	9.74	9.74	9.73	9.74	9.74	9.74	9.73	9.74
38	ASP	3.48 ± 0.02	3.50	3.50	3.46	3.46	3.50	3.50	3.46	3.46
40	GLU	5.38 ± 0.01	5.38	5.37	5.39	5.38	5.38	5.37	5.39	5.38
43	LYS	10.31 ± 0.06	10.40	10.29	10.28	10.27	10.40	10.29	10.28	10.27
51	TYR	11.06 ± 0.02	11.07	11.06	11.08	11.03	11.07	11.06	11.08	11.03
53	ARG	13.03 ± 0.05	13.04	12.97	13.10	12.99	13.04	12.97	13.10	12.99
58	ASP	2.69 ± 0.02	2.69	2.66	2.71	2.68	2.69	2.66	2.71	2.68
63	HIS	3.78 ± 0.06	3.84	3.71	3.74	3.82	3.84	3.71	3.74	3.82
64	LYS	9.84 ± 0.05	9.89	9.86	9.86	9.76	9.89	9.86	9.86	9.76
67	HIS	6.28 ± 0.01	6.27	6.28	6.29	6.28	6.27	6.28	6.29	6.28
69	LYS	10.42 ± 0.00	10.42	10.42	10.42	10.42	10.42	10.42	10.42	10.42
73	TYR	12.31 ± 0.05	12.26	12.27	12.35	12.35	12.26	12.27	12.35	12.35
77	LYS	9.02 ± 0.01	9.03	9.03	9.01	9.02	9.03	9.03	9.01	9.02
79	ARG	12.85 ± 0.04	12.84	12.89	12.80	12.87	12.84	12.89	12.80	12.87
92	TYR	14.17 ± 0.06	14.10	14.13	14.22	14.24	14.10	14.13	14.22	14.24
96	ASP	2.93 ± 0.04	2.98	2.89	2.90	2.95	2.98	2.89	2.90	2.95
108	TYR	10.82 ± 0.01	10.81	10.82	10.83	10.82	10.81	10.82	10.83	10.82
109	ASP	3.41 ± 0.02	3.41	3.39	3.43	3.41	3.41	3.39	3.43	3.41
110	TYR	12.48 ± 0.02	12.48	12.46	12.47	12.50	12.48	12.46	12.47	12.50
113	TYR	10.30 ± 0.00	10.30	10.30	10.30	10.30	10.30	10.30	10.30	10.30
120	ARG	12.03 ± 0.01	12.02	12.03	12.04	12.03	12.02	12.03	12.04	12.03
122	TYR	12.22 ± 0.00	12.23	12.22	12.22	12.22	12.23	12.22	12.22	12.22
123	LYS	10.60 ± 0.02	10.63	10.58	10.58	10.59	10.63	10.58	10.58	10.59
126	LYS	10.37 ± 0.02	10.39	10.35	10.35	10.38	10.39	10.35	10.35	10.38
127	ARG	11.47 ± 0.02	11.45	11.46	11.49	11.48	11.45	11.46	11.49	11.48

ID	Name	Mean±SD	A	B	C	D	E	F	G	H
129	ASP	4.19 ± 0.01	4.20	4.20	4.18	4.19	4.20	4.20	4.17	4.19
131	ARG	12.69 ± 0.00	12.68	12.69	12.69	12.69	12.68	12.69	12.69	12.69
133	TYR	12.48 ± 0.07	12.46	12.55	12.39	12.54	12.45	12.55	12.39	12.54
134	GLU	3.86 ± 0.01	3.87	3.86	3.84	3.86	3.87	3.86	3.84	3.86
135	GLU	3.26 ± 0.01	3.25	3.27	3.28	3.25	3.25	3.27	3.28	3.25
137	TYR	11.44 ± 0.01	11.44	11.43	11.43	11.46	11.44	11.43	11.43	11.46
138	TYR	10.12 ± 0.01	10.12	10.12	10.13	10.11	10.12	10.12	10.13	10.11
139	HIS	6.27 ± 0.00	6.27	6.27	6.27	6.27	6.27	6.27	6.27	6.27
140	ARG	13.81 ± 0.01	13.81	13.81	13.80	13.82	13.81	13.81	13.80	13.82
144	ARG	12.14 ± 0.01	12.14	12.14	12.16	12.14	12.14	12.14	12.16	12.14
146	ASP	3.08 ± 0.02	3.10	3.09	3.05	3.08	3.10	3.09	3.05	3.08
150	HIS	6.45 ± 0.02	6.45	6.44	6.41	6.47	6.46	6.44	6.42	6.47
152	ARG	11.27 ± 0.01	11.28	11.28	11.27	11.26	11.28	11.28	11.27	11.26
156	TYR	10.19 ± 0.04	10.12	10.22	10.22	10.20	10.12	10.22	10.22	10.20
159	LYS	10.01 ± 0.09	9.98	10.14	9.92	9.98	9.98	10.14	9.92	9.98
161	ARG	11.52 ± 0.16	11.45	11.45	11.78	11.41	11.45	11.45	11.78	11.41
169	HIS	4.76 ± 0.02	4.75	4.76	4.78	4.74	4.75	4.76	4.78	4.74
173	GLU	3.59 ± 0.05	3.56	3.58	3.67	3.54	3.56	3.58	3.67	3.54
175	HIS	7.59 ± 0.02	7.61	7.60	7.58	7.56	7.61	7.60	7.58	7.56
178	LYS	10.35 ± 0.05	10.27	10.38	10.38	10.38	10.27	10.38	10.38	10.38
179	C-	3.34 ± 0.02	3.34	3.31	3.35	3.35	3.34	3.31	3.35	3.35

SUPPLEMENTARY TABLE S6: H++-PREDICTED PKA VALUES FOR FRAC.

TABLE S6: H++ predicted pKa values for FraC.

ID	Name	Mean±SD	A	B	C	D	E	F	G	H
4	N+	6.80 ± 0.04	6.78	6.77	6.76	6.83	6.78	6.78	6.83	6.88
10	ASP	4.11 ± 0.04	4.13	4.16	4.14	4.11	4.13	4.14	4.02	4.08
17	ASP	4.74 ± 0.10	4.89	4.73	4.70	4.69	4.89	4.74	4.68	4.63
20	LYS	10.23 ± 0.06	10.15	10.27	10.22	10.28	10.16	10.29	10.21	10.28
24	GLU	5.42 ± 0.10	5.26	5.46	5.49	5.48	5.25	5.47	5.45	5.47
30	LYS	10.41 ± 0.05	10.38	10.41	10.46	10.36	10.35	10.47	10.45	10.37
31	ARG	12.50 ± 0.06	12.49	12.49	12.52	12.46	12.43	12.62	12.53	12.44
32	LYS	10.38 ± 0.03	10.35	10.41	10.39	10.38	10.39	10.42	10.34	10.39
38	ASP	4.94 ± 0.07	4.96	4.88	5.11	4.93	4.95	4.89	4.89	4.92
40	GLU	5.13 ± 0.08	5.11	5.04	5.20	5.07	5.11	5.09	5.28	5.16
43	LYS	9.43 ± 0.20	9.74	9.27	9.37	9.26	9.73	9.32	9.40	9.33
51	TYR	12.27 ± 0.11	12.19	12.18	12.39	12.29	12.34	12.21	12.42	12.12
53	ARG	11.40 ± 0.11	11.38	11.27	11.49	11.36	11.57	11.35	11.28	11.47
58	ASP	4.92 ± 0.08	4.99	5.04	4.85	4.95	4.81	4.93	4.87	4.88
63	HID	6.78 ± 0.33	6.25	7.06	7.00	6.71	6.32	7.04	7.05	6.81
64	LYS	10.34 ± 0.02	10.33	10.37	10.32	10.37	10.34	10.33	10.32	10.34
67	HIS	4.75 ± 0.11	4.68	4.66	4.74	4.72	4.70	4.71	4.99	4.85
69	LYS	10.40 ± 0.02	10.40	10.39	10.43	10.41	10.42	10.39	10.37	10.39
73	TYR	13.45 ± 0.64	13.78	13.46	13.77	13.40	14.25	13.85	12.81	12.23
77	LYS	11.90 ± 0.18	12.20	11.93	11.73	11.77	11.72	12.12	11.93	11.83
79	ARG	12.05 ± 0.09	12.12	12.04	12.01	12.08	12.02	12.02	11.90	12.20
92	TYR	15.73 ± 0.10	15.61	15.74	15.79	15.90	15.60	15.78	15.66	15.79
96	ASP	4.38 ± 0.04	4.37	4.41	4.43	4.41	4.35	4.43	4.32	4.34
108	TYR	13.46 ± 0.31	13.28	13.27	13.10	13.88	13.19	13.37	13.78	13.79
109	ASP	3.50 ± 0.08	3.53	3.42	3.50	3.52	3.50	3.47	3.65	3.38
110	TYR	15.83 ± 0.15	15.79	15.93	15.96	15.88	15.75	15.83	15.99	15.53
113	TYR	9.53 ± 0.05	9.58	9.53	9.55	9.48	9.52	9.59	9.55	9.46
120	ARG	12.03 ± 0.18	12.05	12.21	12.25	12.07	12.06	11.91	12.02	11.68
122	TYR	12.64 ± 0.07	12.62	12.65	12.77	12.62	12.63	12.57	12.72	12.58
123	LYS	10.35 ± 0.07	10.25	10.38	10.38	10.45	10.23	10.38	10.38	10.37
126	LYS	10.45 ± 0.03	10.45	10.44	10.51	10.44	10.42	10.45	10.50	10.43
127	ARG	12.42 ± 0.12	12.20	12.42	12.41	12.46	12.32	12.52	12.56	12.50

ID	Name	Mean±SD	A	B	C	D	E	F	G	H
129	ASP	3.80 ± 0.08	3.77	3.79	3.87	3.79	3.79	3.96	3.76	3.69
131	ARG	11.72 ± 0.04	11.74	11.71	11.76	11.70	11.74	11.65	11.77	11.70
133	TYR	10.80 ± 0.30	10.93	11.00	11.07	10.43	11.10	10.54	10.95	10.40
134	GLU	4.02 ± 0.06	4.05	4.05	3.97	4.07	4.08	4.02	3.91	4.00
135	GLU	4.84 ± 0.07	4.82	4.84	4.89	4.78	4.83	4.72	4.96	4.86
137	TYR	10.35 ± 0.27	10.27	10.48	10.28	9.76	10.41	10.67	10.53	10.43
138	TYR	9.89 ± 0.10	9.99	9.99	9.95	9.76	9.97	9.78	9.80	9.85
139	HIS	6.30 ± 0.02	6.31	6.31	6.30	6.29	6.31	6.26	6.30	6.28
140	ARG	11.88 ± 0.08	11.94	11.90	11.99	11.85	11.93	11.80	11.87	11.74
144	ARG	11.99 ± 0.10	12.10	12.04	12.07	11.98	12.09	11.89	11.96	11.82
146	ASP	5.79 ± 0.16	5.82	5.83	5.84	5.67	5.78	6.10	5.74	5.54
150	HID	7.19 ± 0.14	7.22	7.14	7.22	6.99	7.16	7.38	7.35	7.02
152	ARG	12.49 ± 0.15	12.56	12.53	12.65	12.51	12.56	12.14	12.48	12.50
156	TYR	11.16 ± 0.26	11.50	11.00	11.09	11.41	11.48	11.07	10.89	10.84
159	LYS	10.17 ± 0.14	10.08	10.03	10.18	10.24	10.39	9.98	10.18	10.29
161	ARG	12.30 ± 0.36	12.50	12.37	12.12	11.83	12.44	12.99	12.16	12.01
169	HIS	6.61 ± 0.10	6.57	6.57	6.50	6.54	6.53	6.76	6.71	6.70
173	GLU	5.02 ± 0.11	5.10	5.00	5.12	4.88	5.07	5.14	5.01	4.85
175	HIS	5.66 ± 0.12	5.72	5.57	5.84	5.50	5.70	5.73	5.52	5.72
178	LYS	10.15 ± 0.04	10.12	10.19	10.13	10.19	10.11	10.19	10.13	10.17
179	C-	4.18 ± 0.08	4.11	4.28	4.12	4.25	4.13	4.30	4.10	4.17

SUPPLEMENTARY TABLE S7: PROPKA3 AND H++ COMPARISON OF PKA VALUES FOR FRAC.

TABLE S7: Comparison of pK_a values between PROPKA3 and H++

ID	Residue	PROPKA3 ± SD	H++ ± SD	$\lvert\Delta\rvert$
10	ASP	4.25 ± 0.14	4.11 ± 0.04	0.14
17	ASP	3.76 ± 0.50	4.74 ± 0.10	0.99
20	LYS	10.98 ± 0.33	10.23 ± 0.06	0.74
24	GLU	4.55 ± 0.07	5.42 ± 0.10	0.86
30	LYS	10.25 ± 0.01	10.41 ± 0.05	0.16
31	ARG	11.44 ± 0.01	12.50 ± 0.06	1.06
32	LYS	9.74 ± 0.00	10.38 ± 0.03	0.65
38	ASP	3.48 ± 0.02	4.94 ± 0.07	1.46
40	GLU	5.38 ± 0.01	5.13 ± 0.08	0.25
43	LYS	10.31 ± 0.06	9.43 ± 0.20	0.88
51	TYR	11.06 ± 0.02	12.27 ± 0.11	1.21
53	ARG	13.03 ± 0.05	11.40 ± 0.11	1.63
58	ASP	2.69 ± 0.02	4.92 ± 0.08	2.23
64	LYS	9.84 ± 0.05	10.34 ± 0.02	0.50
67	HIS	6.28 ± 0.01	4.75 ± 0.11	1.53
69	LYS	10.42 ± 0.00	10.40 ± 0.02	0.02
73	TYR	12.31 ± 0.05	13.45 ± 0.64	1.14
77	LYS	9.02 ± 0.01	11.90 ± 0.18	2.88
79	ARG	12.85 ± 0.04	12.05 ± 0.09	0.80
92	TYR	14.17 ± 0.06	15.73 ± 0.10	1.56
96	ASP	2.93 ± 0.04	4.38 ± 0.04	1.45
108	TYR	10.82 ± 0.01	13.46 ± 0.31	2.64
109	ASP	3.41 ± 0.02	3.50 ± 0.08	0.09
110	TYR	12.48 ± 0.02	15.83 ± 0.15	3.35
113	TYR	10.30 ± 0.00	9.53 ± 0.05	0.77
120	ARG	12.03 ± 0.01	12.03 ± 0.18	0.00
122	TYR	12.22 ± 0.00	12.64 ± 0.07	0.42
123	LYS	10.60 ± 0.02	10.35 ± 0.07	0.24
126	LYS	10.37 ± 0.02	10.45 ± 0.03	0.09
127	ARG	11.47 ± 0.02	12.42 ± 0.12	0.95
129	ASP	4.19 ± 0.01	3.80 ± 0.08	0.39
131	ARG	12.69 ± 0.00	11.72 ± 0.04	0.97

ID	Residue	PROPKA3 ± SD	H++ ± SD	$\lvert\Delta\rvert$
133	TYR	12.48 ± 0.07	10.80 ± 0.30	1.68
134	GLU	3.86 ± 0.01	4.02 ± 0.06	0.16
135	GLU	3.26 ± 0.01	4.84 ± 0.07	1.58
137	TYR	11.44 ± 0.01	10.35 ± 0.27	1.09
138	TYR	10.12 ± 0.01	9.89 ± 0.10	0.23
139	HIS	6.27 ± 0.00	6.30 ± 0.02	0.03
140	ARG	13.81 ± 0.01	11.88 ± 0.08	1.93
144	ARG	12.14 ± 0.01	11.99 ± 0.10	0.15
146	ASP	3.08 ± 0.02	5.79 ± 0.16	2.71
152	ARG	11.27 ± 0.01	12.49 ± 0.15	1.22
156	TYR	10.19 ± 0.04	11.16 ± 0.26	0.97
159	LYS	10.01 ± 0.09	10.17 ± 0.14	0.17
161	ARG	11.52 ± 0.16	12.30 ± 0.36	0.78
169	HIS	4.76 ± 0.02	6.61 ± 0.10	1.85
173	GLU	3.59 ± 0.05	5.02 ± 0.11	1.43
175	HIS	7.59 ± 0.02	5.66 ± 0.12	1.93
178	LYS	10.35 ± 0.05	10.15 ± 0.04	0.20

SUPPLEMENTARY REFERENCES

[S1] William Humphrey, Andrew Dalke, Klaus Schulten, et al. Vmd: visual molecular dynamics. Journal of molecular graphics, 14(1):33–38, 1996.

[S2] Schrödinger, LLC. The PyMOL molecular graphics system, version 3.1. June 2025.

[S3] Eric F Pettersen, Thomas D Goddard, Conrad C Huang, Gregory S Couch, Daniel M Greenblatt, Elaine C Meng, and Thomas E Ferrin. Ucsf chimera—a visualization system for exploratory research and analysis. Journal of computational chemistry, 25(13):1605–1612, 2004.

[S4] Elaine C Meng, Thomas D Goddard, Eric F Pettersen, Greg S Couch, Zach J Pearson, John H Morris, and Thomas E Ferrin. Ucsf chimerax: Tools for structure building and analysis. Protein Science, 32(11):e4792, 2023.

[S5] Josh Abramson, Jonas Adler, Jack Dunger, Richard Evans, Tim Green, Alexander Pritzel, Olaf Ronneberger, Lindsay Willmore, Andrew J Ballard, Joshua Bambrick, et al. Accurate structure prediction of biomolecular interactions with alphafold 3. Nature, 630(8016):493–500, 2024.

[S6] Parveen Goyal, Petya V Krasteva, Nani Van Gerven, Francesca Gubellini, Imke Van den Broeck, Anastassia Troupiotis-Tsaïlaki, Wim Jonckheere, Gérard Péhau-Arnaudet, Jerome S Pinkner, Matthew R Chapman, et al. Structural and mechanistic insights into the bacterial amyloid secretion channel csgg. Nature, 516(7530):250, 2014.

[S7] Hang Liu, Rongliang Wang, Dejian Gu, Shengwei Tan, Hongwen Wu, and Quanjun Liu. Expression and purification of a novel mycobacterial porin mspa mutant in e. coli. Journal of Nanoscience and Nanotechnology, 17(12):9125–9129, 2017.

[S8] Mikhail A Lomize, Andrei L Lomize, Irina D Pogozheva, and Henry I Mosberg. Opm: orientations of proteins in membranes database. Bioinformatics, 22(5):623–625, 2006.

[S9] Roland L. Dunbrack and Martin Karplus. Backbone-dependent rotamer library for proteins application to side-chain prediction. Journal of Molecular Biology, 230(2):543–574, 1993.

[S10] Mats HM Olsson, Chresten R Søndergaard, Michal Rostkowski, and Jan H Jensen. Propka3: consistent treatment of internal and surface residues in empirical p k a predictions. Journal of chemical theory and computation, 7(2):525–537, 2011.

[S11] Ramu Anandakrishnan, Boris Aguilar, and Alexey V Onufriev. H++ 3.0: automating p k prediction and the preparation of biomolecular structures for atomistic molecular modeling and simulations. Nucleic acids research, 40(W1):W537–W541, 2012.

[S12] Langzhou Song, Michael R Hobaugh, Christopher Shustak, Stephen Cheley, Hagan Bayley, J Eric Gouaux, and o others. Structure of staphylococcal alpha-hemolysin, a heptameric transmembrane pore. Science, 274(5294):1859–1865, 1996.

[S13] Koji Tanaka, Jose MM Caaveiro, Koldo Morante, Juan Manuel González-Mañas, and Kouhei Tsumoto. Structural basis for self-assembly of a cytolytic pore lined by protein and lipid. Nature communications, 6:6337, 2015.

[S14] Nathan A Baker, David Sept, Simpson Joseph, Michael J Holst, and J Andrew McCammon. Electrostatics of nanosystems: application to microtubules and the ribosome. Proceedings of the National Academy of Sciences, 98(18):10037–10041, 2001.

[S15] Elizabeth Jurrus, Dave Engel, Keith Star, Kyle Monson, Juan Brandi, Lisa E. Felberg, David H. Brookes, Leighton Wilson, Jiahui Chen, Karina Liles, Minju Chun, Peter Li, David W. Gohara, Todd Dolinsky, Robert Konecny, David R. Koes, Jens Erik Nielsen, Teresa Head-Gordon, Weihua Geng, Robert Krasny, Guo-Wei Wei, Michael J. Holst, J. Andrew McCammon, and Nathan A. Baker. Improvements to the apbs biomolecular solvation software suite. Protein Science, 27(1):112–128, 2018.

[S16] Mikhail A Lomize, Irina D Pogozheva, Hyeon Joo, Henry I Mosberg, and Andrei L Lomize. Opm database and ppm web server: resources for positioning of proteins in membranes. Nucleic acids research, 40(D1):D370–D376, 2012.

[S17] Michael Faller, Michael Niederweis, and Georg E Schulz. The structure of a mycobacterial outer-membrane channel. Science, 303(5661):1189–1192, 2004.

[S18] Jeffrey R Wagner, Jesper Sørensen, Nathan Hensley, Celia Wong, Clare Zhu, Taylor Perison, and Rommie E Amaro. Povme 3.0: software for mapping binding pocket flexibility. Journal of chemical theory and computation, 13(9):4584–4592, 2017.
